# A proteomic signature of oocyte quality from models of varying oocyte developmental competence

**DOI:** 10.1093/humrep/deag134

**Published:** 2026-08-20

**Authors:** Emily R Frost, Dulama Richani, Anne Poljak, Ananya Vuyyuru, Xiuhua Liao, Elise Georgiou, J M Binuri Gunasekara, Bettina P Mihalas, Irene E Sucquart, Kaushiki Kadam, Lindsay E Wu, Robert B Gilchrist

**Affiliations:** Fertility & Research Centre, Discipline of Women’s Health, School of Clinical Medicine, University of New South Wales Sydney, Sydney, NSW, Australia; Fertility & Research Centre, Discipline of Women’s Health, School of Clinical Medicine, University of New South Wales Sydney, Sydney, NSW, Australia; Bioanalytical Mass Spectrometry Facility, Mark Wainwright Analytical Centre, University of New South Wales Sydney, Sydney, NSW, Australia; Fertility & Research Centre, Discipline of Women’s Health, School of Clinical Medicine, University of New South Wales Sydney, Sydney, NSW, Australia; Fertility & Research Centre, Discipline of Women’s Health, School of Clinical Medicine, University of New South Wales Sydney, Sydney, NSW, Australia; Fertility & Research Centre, Discipline of Women’s Health, School of Clinical Medicine, University of New South Wales Sydney, Sydney, NSW, Australia; Fertility & Research Centre, Discipline of Women’s Health, School of Clinical Medicine, University of New South Wales Sydney, Sydney, NSW, Australia; Fertility & Research Centre, Discipline of Women’s Health, School of Clinical Medicine, University of New South Wales Sydney, Sydney, NSW, Australia; Fertility & Research Centre, Discipline of Women’s Health, School of Clinical Medicine, University of New South Wales Sydney, Sydney, NSW, Australia; Fertility & Research Centre, Discipline of Women’s Health, School of Clinical Medicine, University of New South Wales Sydney, Sydney, NSW, Australia; School of Biomedical Sciences, University of New South Wales Sydney, Sydney, NSW, Australia; Fertility & Research Centre, Discipline of Women’s Health, School of Clinical Medicine, University of New South Wales Sydney, Sydney, NSW, Australia

**Keywords:** capacitation-IVM (CAPA-IVM), in vitro maturation (IVM), cumulus cells, developmental competence, folliculogenesis, assisted reproductive technologies, proteomics, mass spectrometry

## Abstract

**STUDY QUESTION:**

Which proteins underpin oocyte developmental competence, as modelled by oocytes of variable competence matured *in vivo* or matured *in vitro* under different conditions (capacitation in vitro maturation (CAPA-IVM) or standard IVM)?

**SUMMARY ANSWER:**

Significant differences in the global proteome were observed in both oocytes and their corresponding cumulus cells depending on the mode of oocyte maturation, with key variations in eukaryotic translation, autophagy, and endocytosis pathways within oocytes, and changes in reactive oxygen species detoxification and serine biosynthesis in cumulus cells.

**WHAT IS KNOWN ALREADY:**

Within the ovarian follicle, mammalian oocytes must acquire the necessary molecular machinery to support successful fertilization and embryonic development. Close contact with the surrounding cumulus cells ensures coordinated nuclear and cytoplasmic maturation of the oocyte, along with the accumulation of proteins stored within the oocyte in cytoplasmic lattices and endo-lysosomal vesicular assemblies.

**STUDY DESIGN, SIZE, DURATION:**

This basic science study utilized a mouse model to assess proteomic changes across three oocyte competence models. Key proteins identified in mouse oocytes were also assessed in discarded immature human germinal vesicle (GV) oocytes and MII oocytes following rescue-IVM. Three oocyte maturation methods were tested: (i) *in vivo* maturation, (ii) CAPA-IVM, and (iii) standard IVM. *In vivo* maturation served as a positive control group, whereby metaphase II (MII) mature oocytes were collected from mice stimulated with pregnant mare serum gonadotropin (PMSG) and triggered with hCG, simulating full ovarian stimulation. For the IVM groups, immature cumulus–oocyte complexes (COCs) were collected from mildly stimulated (23 h PMSG) mice. For the standard IVM group, immature COCs were matured in media containing amphiregulin and epiregulin for 18 h. For the CAPA group, COCs were held for 24 h in pre-IVM conditions in the presence of c-type natriuretic peptide (CNP), oestradiol, insulin, and FSH, and then matured via IVM in media containing FSH, amphiregulin, and epiregulin. Four biological replicates were performed for mouse proteomics experiments; three biological replicates were performed for mouse immunocytochemistry experiments; and six replicates were performed for embryology experiments.

**PARTICIPANTS/MATERIALS, SETTINGS, METHODS:**

Four- to six-week-old C57BL/6JAusb mice were used for all mouse experiments. Embryology outcomes were used to confirm the variation in oocyte developmental competence between the three maturation groups. For the *in vivo*, CAPA and IVM groups, mature MII COCs were collected and separated into oocytes and cumulus cells. Oocytes and cumulus cells were subjected to mass spectrometry and bioinformatic analysis was performed using Proteome Discoverer and Ingenuity Pathway Analysis, with data validated by immunofluorescence. To assess conservation of proteins in human oocytes, 49 oocytes were collected from 36 patients following ART cycles and subject to immunofluorescence. Rescue-IVM was also performed with half of the human oocyte cohort to obtain MII oocytes.

**MAIN RESULTS AND THE ROLE OF CHANCE:**

Proteomic profiling (at least two peptides per protein) identified around 1600 proteins in mouse oocytes and 3100 proteins in mouse cumulus cells across all three treatment groups. Differential expression analysis and pattern analysis collectively revealed a signature of proteins that were consistently differentially expressed between *in vivo* and *in vitro* oocyte maturation systems (log2FC of ± 1 and a *P*-value ≤ 0.05). These subsets of proteins were mapped to biological processes including eukaryotic translation, autophagy, and endocytosis pathways within oocytes. Orthogonal validation of clathrin, ribosomal protein L24, and eukaryotic initiation factor 2A supported the proteomic findings, and their expression was conserved in human GV and MII oocytes. Changes in reactive oxygen species detoxification and serine biosynthesis were observed in mouse cumulus cells, with fluorescence intensity changes in ferredoxin-1 and phosphoglycerate dehydrogenase supporting the dysregulation of cumulus cell processes during IVM.

**LARGE SCALE DATA:**

The mass spectrometry data are available via ProteomeXchange with identifier PXD073269.

**LIMITATIONS, REASONS FOR CAUTION:**

The foundational mechanisms of oocyte developmental competence remain elusive, particularly in humans where MII oocytes are heterogenous in quality within the same stimulation cycle and patient. Limitations in this study include the detection and proteome coverage of poorly expressed proteins, the inability to check molecular conservation in human CAPA-IVM and IVM oocytes, the use of fetuin in the mouse *in vitro* systems, and the potential source of keratin proteins in oocyte samples, and these factors must all be carefully considered. C57Bl6/J mice were used as the model species, allowing precise control over differing models of oocyte quality and capacity to analyse large numbers of oocytes. However, care is required when interpreting the significance of these findings in mice to mechanisms regulating human oocyte quality. Nonetheless, the *in vivo* stimulation and both IVM protocols used in this study are clinically relevant and developmentally matched. This study has also not addressed oocyte developmental competence in gonadotropin-free IVM oocytes, which is now a clinical reality.

**WIDER IMPLICATIONS OF THE FINDINGS:**

This study adds to the wider literature indicating that oocytes matured *in vitro*, either through CAPA-IVM or IVM, are unable to achieve the developmental competence rates observed with *in vivo* stimulation. Through examination of the global proteome in oocytes, molecular pathways including eukaryotic translation, autophagy, and endocytosis were dysregulated in *in vitro* oocytes. Recent findings have revealed the critical role of these pathways to developmental competence in the context of *in vivo* development. In cumulus cells, changes in reactive oxygen species detoxification and serine biosynthesis were observed, adding to the extensive knowledge around metabolic activity in cumulus cells as a critical facet of oocyte quality. Combined, these data suggest that the necessary processes of protein storage and degradation in oocytes and metabolism in cumulus cells constitute important components of oocyte quality. These processes appear suboptimal in current IVM systems, providing a future research direction to optimize IVM protocols with consideration to these protein pathways.

**FUNDING:**

This study was funded by a National Health and Medical Research Council Investigator Fellowship (APP1023210) awarded to R.B.G. and by a gift from Open Philanthropy.

**DISCLOSURES:**

R.B.G. is a consultant to Dioseve Inc. L.E.W. is a co-founder, shareholder, director, and advisor of Jumpstart Fertility Inc. L.E.W. is also an advisor and shareholder in EdenRoc Sciences, the parent company of Metro Biotech NSW and Metro Biotech, and in Life Biosciences LLC and its daughter companies. His UNSW Industry Scientia position is partly funded by Proto Axiom. All other authors have no competing interests to disclose.

## Introduction

Over many decades, ART treatment strategies have been refined to improve the efficiency of protocols and importantly reduce the time to live birth for patients. Despite this, the global pregnancy rate per aspiration for IVF/ICSI cycles remains around 26% (Chambers *et al.*, 2021). A principal determinant of ART success is the retrieval of a population of high-quality oocytes that can undergo fertilization and support embryo and foetal development. Despite extensive research efforts, it is still unclear what key molecular events constitute a high-quality oocyte. Thus, there are no therapies to improve oocyte quality and clinical assessment of oocytes remains primarily restricted to morphological assessments (Lemseffer *et al.*, 2022; Sciorio *et al.*, 2022). Elucidating a molecular signature of oocyte developmental competence will provide opportunities to further improve ART outcomes and diagnostic methods to quantify gamete potential.

The capacity of an oocyte to undergo fertilization and support embryo development is referred to as oocyte developmental competence. Developmental competence covers a broad range of contributions to oocyte quality. This includes the attainment of all molecular machinery necessary to support the oocyte through meiosis and early embryo development, up to and beyond activation of the embryonic genome (Tan and Dunning, 2022). Oocyte developmental competence is initiated early in folliculogenesis and is continually acquired as the oocyte stores maternal transcripts, proteins, and metabolites before entering a period of transcriptional silence in late folliculogenesis (Li *et al.*, 2010; Zhang *et al.*, 2018). It is widely accepted that several cellular and molecular processes are critical foundations for a healthy oocyte, including: bidirectional oocyte/cumulus cell communication, accumulation of mitochondria and appropriate metabolic activity, storage of maternal proteins and RNAs, the capacity to elicit appropriate damage responses, resumption of the meiotic cell cycle, proper chromosome condensation, spindle assembly, and faithful chromosome segregation (reviewed in Conti and Franciosi, 2018). Recent studies have highlighted that the efficient removal of unwanted maternal products is also a key aspect of oocyte developmental competence (Sha *et al.*, 2020; Zaffagnini *et al.*, 2024, 2025). Importantly, all these processes must be precisely controlled to ensure coordinated nuclear and cytoplasmic maturation, and this heavily relies on signalling from the associated cumulus cells (Richani *et al.*, 2021). Cumulus cells play a vital role in supplying metabolites, nutrients, and other paracrine signalling molecules to the oocyte, with disruptions in communication and function resulting in poor quality oocytes and decreased rates of embryo formation and development (Hashimoto *et al.*, 1998; Geshi *et al.*, 2000; Fatehi *et al.*, 2002; Richani *et al.*, 2019; Dvoran *et al.*, 2024; Chow *et al.*, 2025; Wang *et al.*, 2025). Despite an ever-expanding knowledge base of the components of oocyte and cumulus cell quality, a molecular signature of a high-quality oocyte remains elusive and other undiscovered processes are likely contributors.

Investigating the underlying mechanisms of human developmental competence is a technical challenge, hence embryo development and pregnancy outcomes remain the gold standard measure for oocyte quality. In humans, interventions to improve oocyte development cannot be readily assessed for developmental competence due to ethical concerns in generating human embryos for research. However, substantial differences in developmental competence exist between oocyte maturation strategies used in clinical ART (Vuong *et al.*, 2020a,b). Collecting human oocytes from antral follicles and maturing them using in vitro maturation (IVM) prior to IVF is a low-intervention approach. Despite successful IVM implementation in the cattle ART industry, human IVM has seen slow clinical progress, primarily attributed to a lower number of resultant embryos and lower cumulative birth rate compared with oocytes from stimulated IVF patients (Walls *et al.*, 2015). A novel biphasic approach to IVM, termed CAPA-IVM, where the oocyte is ‘capacitated’ using a pre-IVM culture step to gain developmental competence *in vitro* before IVM, is an improvement on standard IVM and has been implemented successfully in clinical practice (Sánchez *et al.*, 2017; Sanchez *et al.*, 2019; Vuong *et al.*, 2020b, 2025). While a suitable alternative for women with high antral follicle count or at risk of ovarian hyperstimulation syndrome, cumulative birth rate remains lower for CAPA-IVM patients compared with traditional IVF patients (Vuong *et al.*, 2020a,b, 2025; Gilchrist *et al.*, 2024). These clinical differences in grades of human oocyte developmental competence can be mimicked in a mouse model, where studies have utilized IVM culture systems to investigate the transcriptomic contributions to developmental competence (Dvoran *et al.*, 2024; Wang *et al.*, 2025).

Here, we interrogate constituents of oocyte developmental competence by comparing the proteomic signature of mature mouse metaphase II (MII) cumulus–oocyte complexes (COCs) of differing intrinsic qualities, as matured by various maturation protocols. *In vivo* ovulated MII COCs (termed *in vivo* throughout the manuscript), *in vitro* matured MII COCs (IVM), and MII COCs *in vitro* matured using the CAPA-IVM biphasic IVM system (CAPA) were the chosen protocols, as they are used clinically and importantly display varying levels of developmental competence (Sánchez *et al.*, 2017; Vuong *et al.*, 2020b). We employed label-free mass spectrometry to analyse the proteomes of mouse oocytes and cumulus cells separately. Proteomic profiling identified novel proteins in key biological pathways that are altered between *in vivo* maturation and IVM strategies, revealing a putative protein signature of oocyte developmental competence. Immunofluorescence staining validated components of the proteomic signature and confirmed that these key targets are also conserved in human oocytes.

## Materials and methods

### Human oocyte collection

Approval of this study was granted by the South Eastern Sydney Local Health District Ethics Committee (2021-ETH00018). Informed consent for the donation of discarded oocytes was provided by couples undergoing ICSI at the Fertility & Research Centre in the Royal Hospital for Women, Sydney. Ovarian stimulation was performed with FSH using either GnRH agonist or antagonist protocols, according to standard clinical procedures (Fig. 1A). Following COC collection, denudation, and staging, immature oocytes were identified and labelled as research material. Only immature oocytes that could not be used for treatment and would have otherwise been disposed of were used for this study. This means that the cohort of human oocytes used in this study are poor quality, having not matured in a stimulation cycle. Cells were transported at 37 °C in a hand-held portable incubator (Thermo Cell Transporter 3018, Labotect, Göttingen, Germany) for ∼15 min to the research laboratory and staged as either germinal vesicle (GV) or metaphase-I (MI). A total of 49 oocytes from 36 patients (ranging in age from 18 to 41 years), collected between June 2025 to November 2025, were used in this study.

**Figure 1.**
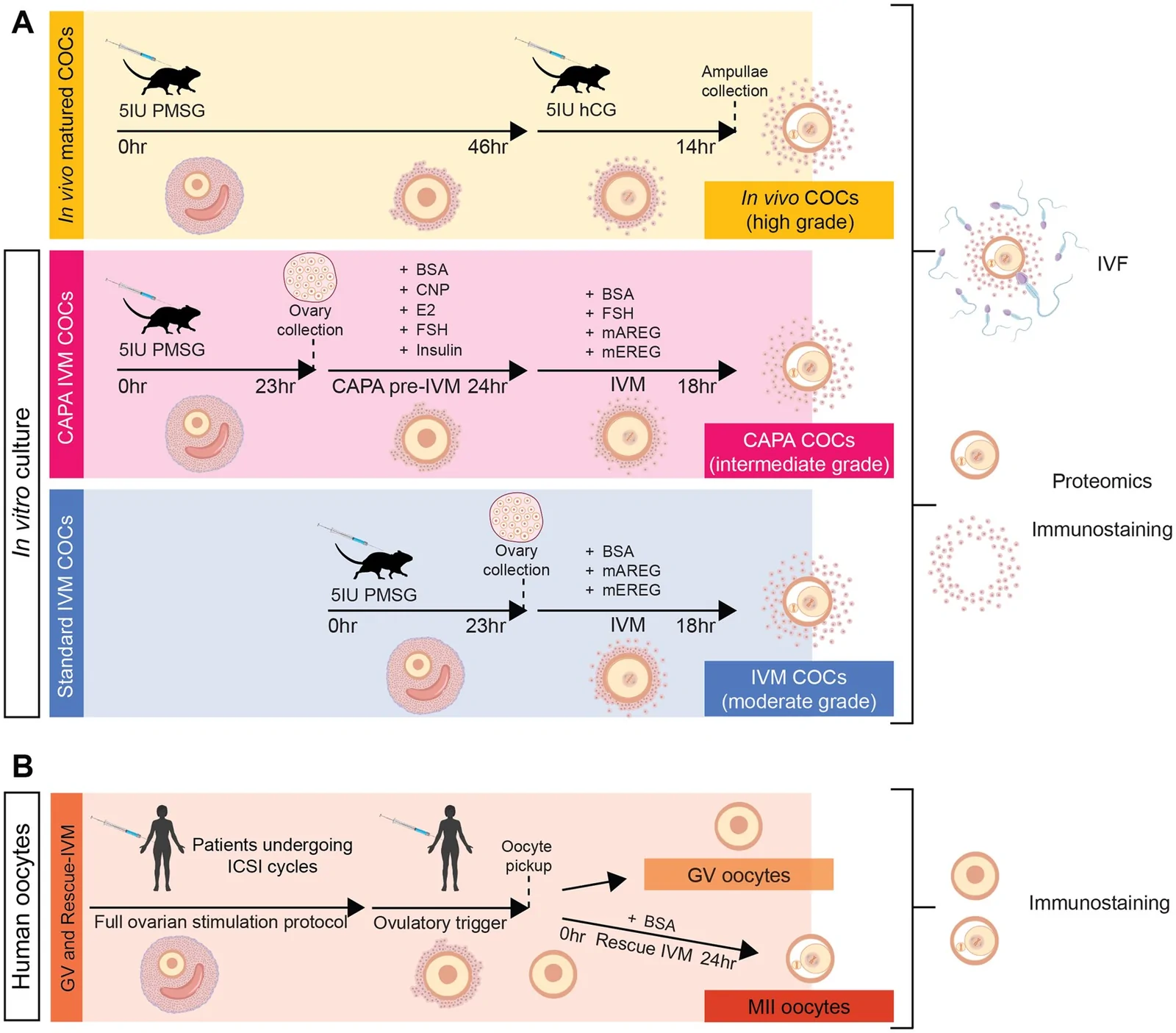
**Schematic showing experimental treatment schedules, ovary collection timepoints, and endpoints assessed.** (**A**) For *in vivo* maturation (yellow box), 4- to 6-week-old female C57Bl/6J mice were stimulated with PMSG for 46 h, triggered with hCG and mature COCs were retrieved 14 h post-trigger. For *in vitro* culture groups, 4- to 6-week-old female C57Bl/6J mice were stimulated with PMSG and ovaries collected 23 h later to retrieve immature COCs. For the CAPA treatment group (pink box) COCs were cultured in CAPA pre-medium for 24 h and then IVM medium containing listed additives for 18 h. For the standard IVM treatment group (blue box), COCs were cultured in IVM medium for 18 h. COCs were then used intact for embryology assessments or separated into oocytes and cumulus cells for proteomics and validation experiments. (**B**) For protein assessment in human oocytes, patients underwent a full stimulation protocol for an ICSI cycle as per standard clinical procedures. Following oocyte retrieval, immature GV oocytes were collected from the clinic and transported to the lab. Oocytes were allocated into two groups, either fixed at the GV stage, or oocytes underwent rescue-IVM for 24 h to reach the MII stage. Immunostaining experiments were performed on both cohorts of oocytes. Icons used in the figure were generated in Biorender. Gilchrist, R. (2026) https://BioRender.com/884hwez. CAPA-IVM, capacitation-IVM; COCs, cumulus–oocyte complexes; GV, germinal vesicle; IVM, in vitro maturation; MII, metaphase II; PMSG, pregnant mare serum gonadotropin.

### Human oocyte maturation

To obtain mature MII human oocytes, GV oocytes were subjected to rescue-IVM (Fig. 1B). GV oocytes were washed three times with standard IVM media containing bicarbonate-buffered alpha minimum essential medium (αMEM) (catalogue 12000063, with custom formulation to replace L-glutamine with L-alanyl-L-glutamine at 2 mM concentration, Thermo Fisher Scientific, Grand Island, NY, USA) + 3 mg/ml fatty-acid-free bovine serum albumin (BSA, catalogue A8806, Sigma-Aldrich, Merck, Darmstadt, Germany) supplemented with 50 μg/ml gentamycin (catalogue G1914, Sigma-Aldrich). Oocytes were then transferred to pre-equilibrated standard IVM medium in 4-well culture dishes (NUNC, Thermo Fisher Scientific, Roskilde, Denmark) and cultured for 24 h in 37 °C, 5% CO_2_ in humidified air. MII oocytes following rescue-IVM were then prepared for immunocytochemistry as detailed below.

### Animals

Four- to six-week-old C57BL/6JAusb female mice were used for all experiments and supplied by Australian BioResources, Mossvale, NSW, Australia. All experiments involving the use of animals were conducted in accordance with the Australian Code of Practice for the Care and Use of Animals for Scientific Purposes. Mice were sustained with a 12-h light-dark cycle, had *ad libitum* access to food and water, and were monitored weekly. All procedures were approved by the UNSW Animal Care & Ethics Committee (ethics numbers 17/105A, 22/40A, and 6531).

### Mouse experimental model and COC collection

This study employed three models of mouse oocyte quality, or oocyte developmental competence (Fig. 1A): (i) high grade oocytes matured *in vivo* following superovulation, (ii) intermediate grade oocytes matured *in vitro* using CAPA-IVM, and (iii) moderate grade oocytes matured *in vitro* using standard IVM. For all treatment groups, four- to six-week-old C57BL/6JAusb female mice were stimulated with 5 IU pregnant mare serum gonadotrophin (PMSG, Folligon; Intervet, Boxmeer, The Netherlands and PMSG; Prospec-Tany Technogene Ltd, Rehovot, Israel). For CAPA-IVM and standard IVM treatment group collections, ovaries were harvested 23 h after PMSG injection (Fig. 1A) and placed in HEPES-buffered αMEM (catalogue 12000063, with custom formulation to replace L-glutamine with L-alanyl-L-glutamine at 2 mM concentration, Thermo Fisher Scientific) containing 3 mg/ml BSA (catalogue A7906, Sigma-Aldrich), 50 μg/ml gentamycin (catalogue G1914, Sigma-Aldrich), and 100 µM 3-isobutyl-1-methylxanthine (IBMX, catalogue I5879, Sigma-Aldrich). Ovarian preovulatory follicles were punctured with a 1 ml insulin syringe attached to a 29-gauge needle to release COCs from the ovary and COCs were gently pipetted into fresh αMEM media + 3 mg/ml BSA + 100 µM 3-isobutyl-1-methylxanthine + 50 μg/ml gentamycin. For *in vivo* matured oocyte collections, at 46 h post-PMSG injection, mice were injected with 5 IU hCG (Chorulon, MSD Animal Health, Bendigo, Australia) to induce ovulation. Ovulated COCs were collected from the oviductal ampullae using a 1 ml insulin syringe attached to a 29-gauge needle and placed into αMEM + 3 mg/ml BSA + 50 μg/ml gentamycin media. To remove cumulus cells from ovulated oocytes, COCs were briefly incubated for 1 min in αMEM medium supplemented with 3 mg/ml BSA and hyaluronidase (catalogue H4272, Sigma-Aldrich).

### Mouse oocyte IVM and pre-IVM

#### CAPA pre-IVM and IVM system

Mouse CAPA-IVM and standard IVM were performed as previously described (Zhao *et al.*, 2020). Following COC collection for the CAPA-IVM treatment group (Fig. 1A), COCs were washed three times in HEPES-buffered αMEM (catalogue 12000063, with custom formulation to replace L-glutamine with L-alanyl-L-glutamine at 2 mM concentration, Thermo Fisher Scientific) + 3 mg/ml BSA media (catalogue A7906, Sigma-Aldrich) to remove the meiotic maturation inhibitor IBMX. CAPA pre-IVM media was prepared with bicarbonate-buffered αMEM + 3 mg/ml BSA supplemented with 50 μg/ml gentamycin (catalogue G1914, Sigma-Aldrich), 2.5 mIU/ml recombinant human FSH (Follitropin Beta; Puregon, Organon, Oss, the Netherlands), 5 ng/ml insulin (catalogue I9278, Sigma-Aldrich), 10 nM estradiol (E2; catalogue E4389, Sigma-Aldrich), and 25 nM c-type natriuretic peptide (CNP, catalogue No. 3520, Tocris Bioscience, Abingdon, UK). For experiments where *in vitro* embryo production was the endpoint, 1 mg/ml fetuin (catalogue F3385, Sigma-Aldrich) was also added to the media to prevent zona pellucida hardening. COCs were then washed three times in the CAPA pre-IVM media, before they were transferred to 500 μl pre-equilibrated CAPA pre-IVM medium (up to 50 COCs per 500 μl of media) in 4-well culture dishes (NUNC, Thermo Scientific) for 24 h in 37 °C, 5% CO_2_ in humidified air. Following CAPA pre-IVM, COCs were washed three times in CAPA-IVM media (bicarbonate-buffered αMEM + 3 mg/ml fatty-acid-free BSA supplemented with 50 μg/ml gentamycin, 2.5 mIU/ml FSH, 50 ng/ml recombinant mouse amphiregulin (mAREG; catalogue No. 989-AR, R&D Systems, Minneapolis, MN, USA), and 50 ng/ml recombinant epiregulin (mEREG; catalogue No. 1068-ER, R&D Systems)). For experiments where *in vitro* embryo production was the endpoint, 1 mg/ml fetuin (catalogue F3385, Sigma-Aldrich) was also added to the IVM media. COCs were then cultured in 500 μl CAPA IVM medium as described earlier for 18 h in 37 °C, 5% CO_2_ in humidified air. Following CAPA-IVM, oocyte maturation was not assessed and COCs were either immediately prepared for IVF, immunocytochemistry or COCs were denuded using a narrow glass pipette, providing separate oocyte and cumulus cell samples. Cells were either processed for immunocytochemistry or snap frozen for proteomics (see below). Figure 1A shows an illustration of the various treatment groups, their culture components, and culture timing.

#### Standard IVM system

Following COC collection for the IVM treatment group (Fig. 1B), COCs were washed three times in HEPES-buffered αMEM + 3 mg/ml BSA media. COCs were then washed three times in standard IVM media, containing bicarbonate-buffered αMEM + 3 mg/ml BSA (catalogue A7906, Sigma-Aldrich) supplemented with 50 μg/ml gentamycin (catalogue G1914, Sigma-Aldrich), 50 ng/ml mAREG (catalogue No. 989-AR, R&D Systems), and 50 ng/ml mEREG (catalogue No. 1068-ER, R&D Systems). For experiments where *in vitro* embryo production was the endpoint, 1 mg/ml fetuin (catalogue F3385, Sigma-Aldrich) was also added. COCs were then cultured in 500 μl standard IVM medium for 18 h in 37 °C, 5% CO_2_ in humidified air. Following IVM, COCs were treated as per CAPA-IVM COCs, as described above (Fig. 1A).

### Mouse *in vitro* embryo production

Performing IVF of COCs after CAPA-IVM, standard IVM or *in vivo* maturation was used as an assessment of oocyte quality, as it provides a measure of preimplantation embryo development. A cohort of 270 C57BL/6JAusb mice was separated into CAPA-IVM, standard IVM, and *in vivo* maturation groups (90 per group) across six replicates. Following CAPA-IVM, standard IVM, or *in vivo* maturation, COCs were fertilized and the resulting embryos were cultured for 6 days as previously described (Stocker *et al.*, 2020). All media (Research Wash, Research Fert and Research Cleave) were purchased from ART Lab Solutions (Adelaide, Australia). Briefly, COCs were washed three times in Research Wash media, supplemented with 4 mg/ml fatty-acid-free BSA, then washed three times in Research Fert media, supplemented with 4 mg/ml fatty-acid-free BSA. Sperm used to fertilize oocytes was collected fresh from hybrid CBB6F1 males > 12 weeks of age and capacitated in Research Fert + BSA for at least 1 h. COCs were co-incubated with capacitated sperm for 4 h at 37 °C with 5% O_2_, 6% CO_2_, and 89% N_2_ in Research Fert media + BSA. Presumptive zygotes were washed three times in Research Wash media + BSA, then washed three times in Research Cleave media, supplemented with 4 mg/ml fatty-acid-free BSA. Embryos were cultured in Research Cleave media + BSA covered by a layer of washed paraffin oil, at a density of one embryo per 2 μl media. Fertilization (percentage of 2-cell embryos per oocyte) and blastocyst formation rates (percentage of blastocyst and hatching blastocysts per cleaved embryo) were assessed at Days 2, 5, and 6 post-fertilization. Blastocyst formation was visually defined by the presence of the blastocoel and inner cell mass structure. Hatching blastocyst was defined by embryonic cells protruding from the zona pellucida.

### Protein extraction and mass spectrometry for proteomic analysis

A cohort of 140 C57BL/6JAusb mice was separated into the three treatment groups across four runs. For each replicate, COCs were pooled from numerous mice following treatment. Four biological replicates, with each containing over 100 COCs/treatment group (*in vivo*, CAPA-IVM, and IVM) were included in the proteomic analysis. Oocytes and cumulus cells were analysed separately. The number of COCs used for each replicate is listed in Supplementary Table S1, but ranges from 106 COCs to 316 COCs. Mass spectrometry methodology and bioinformatics analysis were performed as previously described (Richani *et al.*, 2024). Briefly, cells were lysed and protein extracted in RIPA buffer (containing protease inhibitors; 0.1 mM phenylmethylsulfonyl fluoride and 10 mM EDTA. Samples were run on a QExactive mass spectrometer (Thermo Electron, Bremen, Germany), with peptide separation on a Dionex UltiMate 3000 nano-liquid chromatography (nano-LC) system (Thermo Fisher Scientific), equipped with an autosampler (Dionex, Amsterdam, Netherlands).

### Bioinformatics analysis

Proteome Discoverer 2.4 (Thermo Fisher Scientific) was used to perform database searching of all mass spectrometry raw files for identifying peptides/proteins and quantifying peak abundances (areas) for the purpose of determining protein expression change across treatments. For the processing workflow, the following parameters were used: retention time alignment was performed with a maximum 3 min retention time shift and a minimum signal: noise threshold of 5, use of three search engines (Mascot, Sequest, and MS Amanda), protein level false discovery rate (FDR) was reported based on a concatenated decoy database (FDR set between 1% and 5%), with an identification requirement of a minimum of two peptide sequences per protein. For the consensus workflow, the following parameters were used: precursor quantification based on peak area and use of unique (but not razor) peptides, normalization was based on total peptide amount and protein abundance calculated as summed abundances, and relative quantification was presented as pairwise peak abundance (area) ratios of treatment groups compared to each other. No data imputation was used, and a *t*-test (background-based) was used to determine expression differences. Principal component analysis (PCA) and heatmaps were generated within the Proteome Discoverer 2.4 software. The protein list was exported from Proteome Discoverer 2.4 and refined to include only proteins with a quantitative value in at least three replicates within a treatment group and a minimum of two unique peptides. From this refined list of proteins, differentially expressed proteins were determined by pairwise comparison, with at least a log2FC of ± 1 (i.e. minimum 2-fold expression difference) and a *P*-value ≤ 0.05. Volcano plots of differentially expressed proteins were generated in Excel. The lists of differentially expressed proteins were further interrogated using Ingenuity Pathway Analysis software (Qiagen, Hilden, Germany) to identify canonical pathways and upstream regulators associated with the differentially expressed proteins (Krämer *et al.*, 2014).

### Immunocytochemistry

In total, 120 mice were used to obtain mouse COCs for five antibody stainings across the three treatment groups for three replicates. Mouse COCs and human oocytes were prepared for immunostaining as previously described with minor modifications (Mihalas *et al.*, 2024). Briefly, COCs and oocytes were quickly washed in PHEM buffer (60 mM free acid PIPES, 25 mM free acid HEPES, 10 mM EGTA, and 4 mM magnesium sulphate heptahydrate; pH 6.9) containing 0.25% Triton X-100 at 37 °C, before proceeding with the immunocytochemistry protocol. Following the wash, cells were fixed with 4% formaldehyde in PHEM buffer with 0.25% Triton X-100 for 30 min. Cells were then permeabilized in phosphate-buffered saline with 0.25% Triton X-100 (PBST) for 15 min at room temperature, before blocking with 3% BSA in PBST for 2 h at room temperature. Cells were incubated in primary antibodies diluted in 3% BSA and 7% goat serum in PBST overnight at 4 °C. Antibody details and dilutions are listed in Supplementary Table S2. Following overnight incubation, cells were rinsed two times in 1% BSA in PBST before a 1 h wash in 1% BSA in PBST, before incubation with species-matched secondary antibodies (goat anti-rabbit Alexa Fluor 647 (1:500; A-21244, Thermo Fisher Scientific) and goat anti-rabbit Alexa Fluor 488 (1:500; A-11008, Thermo Fisher Scientific). Secondary antibodies were diluted in 3% BSA and 7% goat serum in PBST and incubated for 2 h at room temperature. Following secondary antibody staining, COCs were co-stained with Alexa Fluor 488 Phalloidin (A12379, Thermo Fisher Scientific) in 1% BSA and 7% donkey serum or 7% goat serum diluted in PBST overnight at 4 °C. Oocytes and COCs were counterstained with the nuclear stain Hoechst (1:2000, H3570, Thermo Fisher Scientific) and mounted on FluoroDishes (FD35-100, WPI, Sarasota, FL, USA) in 0.4% polyvinyl alcohol (PVA, Sigma-Aldrich) in PBS under paraffin oil.

### Confocal microscopy and image analysis

Oocytes and COCs were imaged using the LSM980 laser scanning confocal microscope (Zeiss, Oberkochen, Germany). Images were taken using the 40× oil objective (Zeiss). To prevent emission overlap, images were acquired sequentially using separate, wavelength-sensitive matched detectors and fluorophores with minimal emission spectra overlap were used. Any potential crosstalk was corrected by adjusting the emission spectra wavelengths and sampling parameters satisfied the Nyquist criterion. For oocytes and COCs, single-slice images were taken across the equatorial plane and where possible encompassing the nuclear material within the oocyte. The following lasers were used in combination and isolation for the various staining: 488 and 639 nm. Representative images and fluorescence intensity analyses were performed on a single slice image using the Fiji software (Schindelin *et al.*, 2012). For quantification, treatment groups were imaged on the same day using the same parameters to allow for accurate intensity comparisons. For each replicate, 5–15 individual oocytes or COCs were imaged and analysed. Thresholding (default) was performed to segment regions of interest (ROIs) and analyse the mean grey value and the integrated density (sum of pixel intensity values within a ROI). For cumulus cell analysis, images containing only cumulus cells were analysed and integrated density values were normalized to cell count per image. Cell counts were performed by segmenting images using the Stardist macro (Schmidt *et al.*, 2018) and counting DAPI+ nuclei as ROIs. For oocyte analysis, ROIs were drawn encompassing only the oocyte for analysis. All measurements were collated in Microsoft Excel and graphs were generated in GraphPad Prism 9.5.1 (GraphPad Software, San Diego, CA, USA).

### Statistical analyses

Fertilization rates were calculated as a proportion of the total number of oocytes that were fertilized with sperm. Hatching blastocyst and total blastocyst rates were calculated as a proportion of the total number of cleaved embryos in each treatment group. Fertilization and blastocyst rates were represented in percentages and arcsine transformed before one-way ANOVA with Tukey’s *post hoc* test for statistical analysis. For fluorescence intensity, data were normalized to the mean of the *in vivo* group of each replicate before performing a one-way ANOVA with Tukey’s *post hoc* test for statistical analysis. No randomization was performed on the datasets analysed in this study and confounders were not controlled. Data were analysed using GraphPad Prism 9.5.1 (GraphPad Software) and differences were considered significant when *P* ≤ 0.05.

## Results

### Oocyte developmental competence is compromised *in vitro*

In this study, we utilized three established models that generate oocytes of high, medium, and low quality. Importantly, each system produces mature MII COCs at similar rates; however, there is variable developmental potential observed, measured by the ability to form embryos, with mouse blastocyst rates of ∼84% (*in vivo* (Sanfins *et al.*, 2015)), 60% (CAPA-IVM, (Zhao *et al.*, 2020)), and 27% (IVM, (Sanfins *et al.*, 2015)) in previous studies. The two *in vitro* systems examined here were CAPA-IVM (intermediate grade) and standard IVM (moderate grade), which were compared to high-grade *in vivo*-matured COCs (*in vivo*) (Fig. 1).

To confirm the varying developmental potential of oocytes retrieved in our hands, we assessed embryo development through to Day 6 post-fertilization as a direct measure of oocyte developmental competence (Fig. 2A). No significant differences in 2-cell cleavage rates were observed between *in vivo*, CAPA-IVM, and IVM oocyte groups at Day 2 (Fig. 2B and C). Relative to *in vivo*-matured oocytes, both the CAPA-IVM and IVM groups showed lower rates of blastocyst formation by Day 5 of embryo culture (Fig. 2D and E), a trend which continued at Day 6 (Fig. 2F and H). Blastocyst hatching rates were also lower in CAPA-IVM and IVM groups relative to the *in vivo* group, by 36% and 40%, respectively (Fig. 2G). In contrast to previously published results (Sanfins *et al.*, 2015; Zhao *et al.*, 2020; Gong *et al.*, 2023), no statistically significant differences in blastocyst outcomes were observed between CAPA-IVM and IVM matured oocytes (Fig. 2D, F, and G). In all three cohorts, embryos that cleaved but did not reach the blastocyst stage were either arrested or fragmented. In summary, these data illustrate that oocytes matured *in vitro* show decreased ability to reach the blastocyst stage, and thus reduced developmental competence, when compared with oocytes matured *in vivo*. Importantly, CAPA-IVM and IVM patient cohorts both experience reduced cumulative live birth rates when compared to IVF patients (Walls *et al.*, 2015; Vuong *et al.*, 2020a), providing an opportunity to reveal molecular pathways that could help improve IVM approaches.

**Figure 2.**
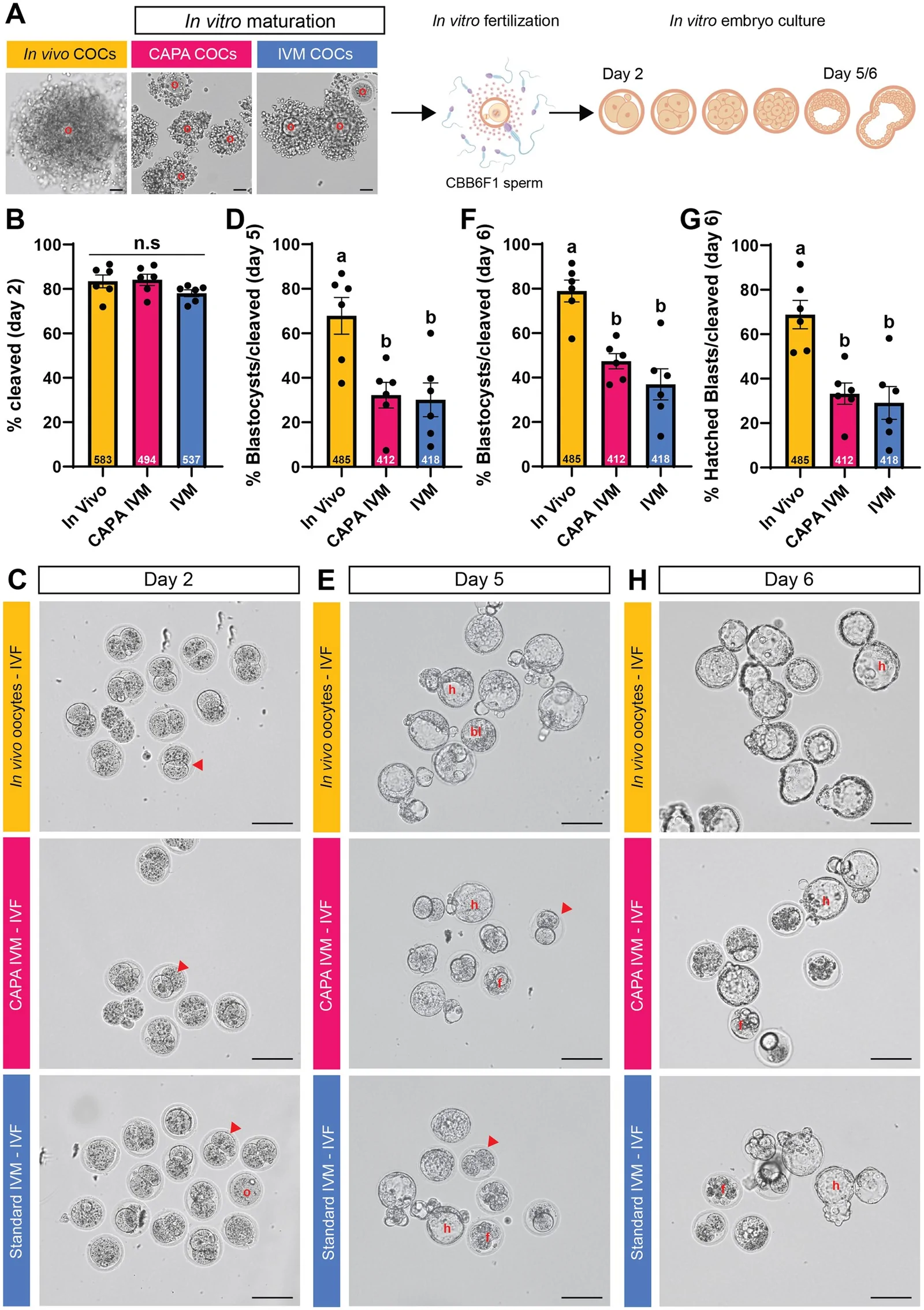
***In vitro* culture decreases oocyte developmental competence as measured by embryo formation and development.** (**A**) Brightfield images of COCs following *in vivo* maturation (yellow), CAPA IVM (pink), and standard IVM (IVM; blue). Red circles mark oocytes, and scale bar is 50 µm. Diagram of IVF followed by morphological assessment of embryos at Days 2, 5, and 6 of *in vitro* embryo culture. (**B**) Fertilization rate (percentage of cleaved embryos per oocyte transferred to culture), (**D**) Day 5 blastocyst rate, (**F**) Day 6 blastocyst rate, and (**G**) Day 6 hatching blastocyst rate (percentage of hatching blastocysts per cleaved embryo) following oocyte maturation. Corresponding images of embryos at Day 2 (**C**), Day 5 (**E**), and Day 6 (**H**) are shown. Red arrowheads mark cleaved embryos, red circles show oocytes that failed to cleave, hatched blastocysts (red h), blastocyst (red bl), and fragmented embryos (red f). Images taken at 10× magnification and scale bar denotes 100 µm. All embryo outcome data were arcsine transformed and one-way ANOVA followed by Tukey’s *post hoc* tests were performed. Numbers above the *x*-axis denote the total number of oocytes (panel B) and total number of cleaved embryos (panel D, F, G) in each oocyte group across the six replicates. Bars with no common superscripts are significantly different (*P* < 0.05). Icons used in the figure were generated in Biorender. Gilchrist, R. (2026) https://BioRender.com/884hwez. CAPA-IVM, capacitation-IVM; COCs, cumulus–oocyte complexes; IVM, in vitro maturation.

### The proteomes of both oocytes and cumulus cells are significantly altered between oocytes of high and low competence

To investigate the molecular mechanisms that underpin the differences between *in vivo* and *in vitro* oocyte developmental competence, we used a label-free proteomic approach to comprehensively profile the proteome in oocytes and their corresponding cumulus cells. In total, we successfully identified 1736 and 3212 proteins in oocyte and cumulus cell samples, respectively, consistent with proteomic coverage obtained previously in other studies (Chen *et al.*, 2023; Sun *et al.*, 2023; Zhang *et al.*, 2023; Richani *et al.*, 2024) (Supplementary Data Files S1 and S2). Within the treatment groups, an average number of 8.5 (*in vivo*), 9.0 (CAPA), and 8.8 (IVM) unique peptides were identified per protein in oocytes, resulting in an average protein coverage of 27.5%, 29.2%, and 28.4%, respectively (Fig. 3A). In cumulus cells, an average number of 9.6 (*in vivo*), 9.8 (CAPA), and 9.8 (IVM) unique peptides were identified per protein, encompassing an average protein coverage of 27.4%, 27.7%, and 27.9%, respectively (Fig. 3B). PCA of global proteome expression revealed diversity in oocyte replicates across principal component 1, however *in vivo*-matured oocytes separated from CAPA-IVM and standard IVM oocytes along principal component 2 (Fig. 3C). Cumulus cells clustered more distinctly in PCA, with *in vivo*-matured cumulus cells segregated to the left on principal component 1 (Fig. 3D). Of the proteins identified in each group (in a minimum of three replicates), most proteins were found in all treatment groups in both oocytes (86.6% overlap) and cumulus cells (92.1% overlap) (Fig. 3E and F). Key oocyte proteins such as zona pellucida sperm-binding proteins 1–3 (ZP1, ZP2, and ZP3) were present in all groups, along with critical oocyte-secreted factors growth/differentiation factor 9 (GDF-9) and bone morphogenetic protein 15 (BMP-15) (Supplementary Fig. S1). In cumulus cells, known marker proteins, including cytochrome P450 11a1 (CYP11A1), insulin-like growth factor 1 receptor (IGF1R), pentraxin-related protein (PTX3), and prostaglandin synthase 2 (PTGS2) were present in all three groups (Supplementary Fig. S1). Other cumulus cell marker proteins including steroidogenic acute regulatory protein (STAR), anti-Müllerian hormone (AMH), and FSH receptor (FSHR) were not detected in any group. Regardless, the comprehensive proteomic coverage across oocyte maturation systems allowed us to delve further into the molecular changes in both oocytes and cumulus cells.

**Figure 3.**
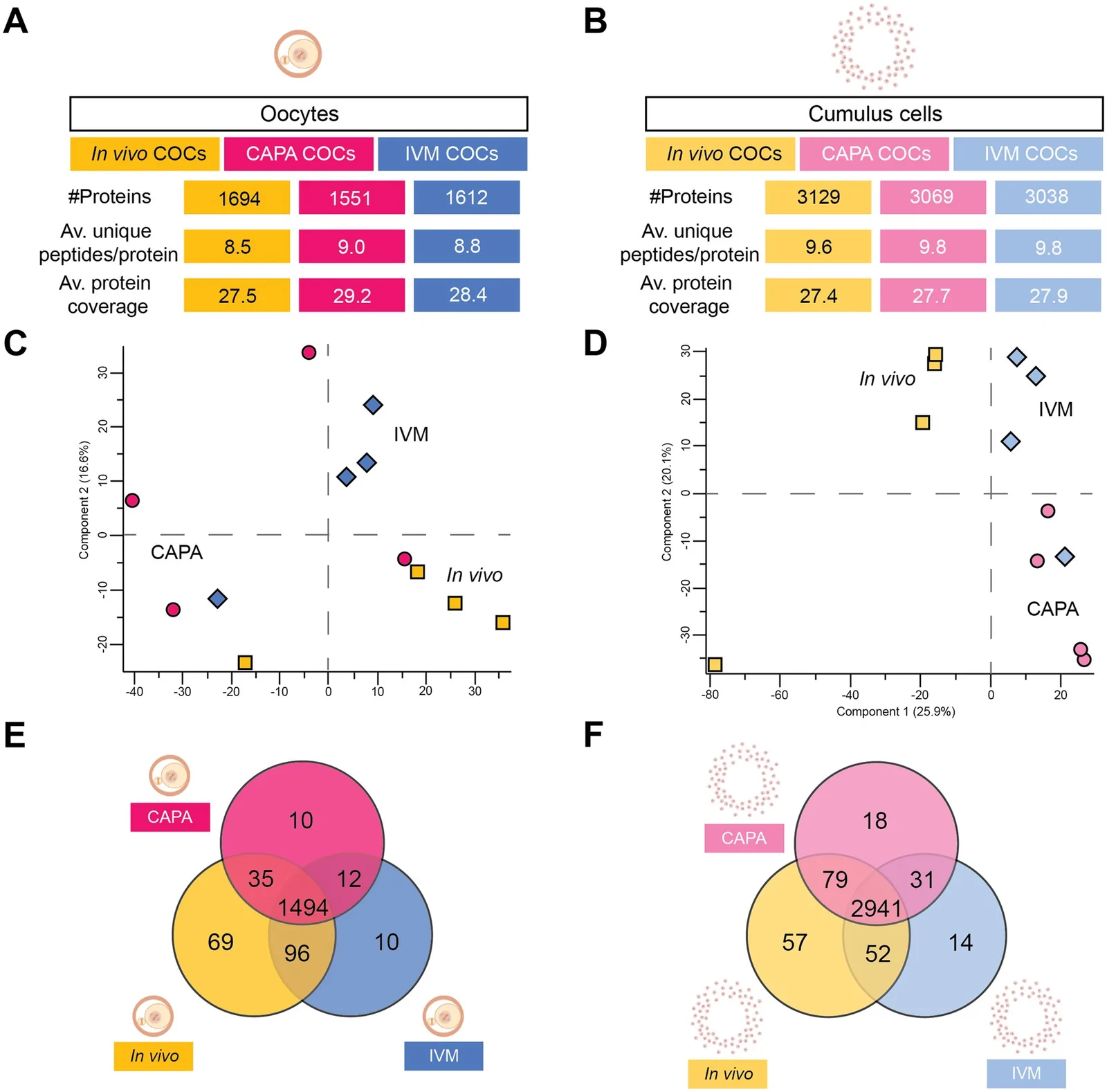
**Proteomic characterization of oocytes and cumulus cells following *in vivo* maturation and IVM.** (**A**) The total number of proteins identified, average number of unique peptides identified/protein and the average protein coverage (%) within oocytes matured *in vivo* (yellow), following CAPA-IVM (pink) and following standard IVM (blue). (**B**) The total number of proteins identified, average number of unique peptides identified/protein, and the average protein coverage (%) within cumulus cells matured *in vivo* (light yellow), following CAPA-IVM (light pink) and following standard IVM (light blue). Principal component analysis (PCA) from each maturation method (*in vivo* (yellow squares), CAPA-IVM (pink circles), and standard IVM (blue diamonds)) for both (**C**) oocytes and (**D**) cumulus cells. (**E**) Venn diagram of shared and unique proteins across the three groups of oocytes. (**F**) Venn diagram of shared and unique proteins across the three groups of cumulus cells. Icons used in the figure were generated in Biorender. Gilchrist, R. (2026) https://BioRender.com/884hwez. CAPA-IVM, capacitation-IVM; IVM, in vitro maturation; PCA, principal component analysis.

### Lower competence oocytes show expression changes in translation machinery and endocytosis proteins

Using pairwise differential expression comparisons to identify differences between oocyte cohorts, we found a small proportion of the total identified proteins (7.2% CAPA vs *in vivo*, 7.2% CAPA vs IVM, and 7.5% IVM vs *in vivo*) were altered across the three models (fold-change ± 2 and *P*-value ≤ 0.05, Fig. 4A, Supplementary Data File S3). To refine our protein list, we focused on differentially expressed proteins that were dysregulated in the same direction in CAPA and IVM compared to the *in vivo* group (log2FC of ± 1, *P*-value ≤ 0.05, Supplementary Data File S4), that reflect the changes we observed in our embryology outcomes and to reveal proteins that are critical for developmental competence. Our analysis revealed a list of 34 proteins differentially expressed in both CAPA-IVM and IVM groups when compared to *in vivo* oocytes (Fig. 4B). Within this list, proteins involved in eukaryotic translation (EIF2A, EIF3M, RPL24, and RPS24), RNA processing (DCP1A, HNRNPAB, HNRNPL, and HNRNPU), and autophagy (CHMP2A and CHMP4B) were consistently altered. Ingenuity Pathway Analysis (IPA) of the pairwise comparisons identified several canonical pathways, with keratinization highly enriched in CAPA-IVM oocytes (Fig. 4C). Keratinization proteins may be present due to contamination from human handling or animal fur fibres from the ovary collection, or may be biologically relevant components of the oocyte cytoskeleton (Lim *et al.*, 2020). Due to protein sequence similarity, we were unable to untangle whether the keratin proteins were of human or mouse origin. In IVM oocytes, cellular stress pathways such as granzyme A signalling, DNA damage, and oxidative phosphorylation were enriched when compared with *in vivo* oocytes (Fig. 4D). Notably, eukaryotic translation initiation, apoptotic execution phase, meiotic synapsis, and nonsense-mediated decay pathways all showed significant enrichment scores in both pairwise comparisons (Fig. 4C and D). Using the same approach, we also analysed the differentially expressed proteins altered in the same direction in CAPA and *in vivo* oocytes compared to the IVM group (log2FC of ± 1, *P*-value ≤ 0.05, Supplementary Fig. S2 and Supplementary Data File S4), to identify protein expression underlying the culture improvements implemented by the biphasic CAPA system. There were 45 proteins differentially expressed between CAPA-IVM and *in vivo* groups when compared to IVM oocytes, with histone proteins and oxidative phosphorylation proteins highlighted by pathway analysis (Supplementary Fig. S2).

**Figure 4.**
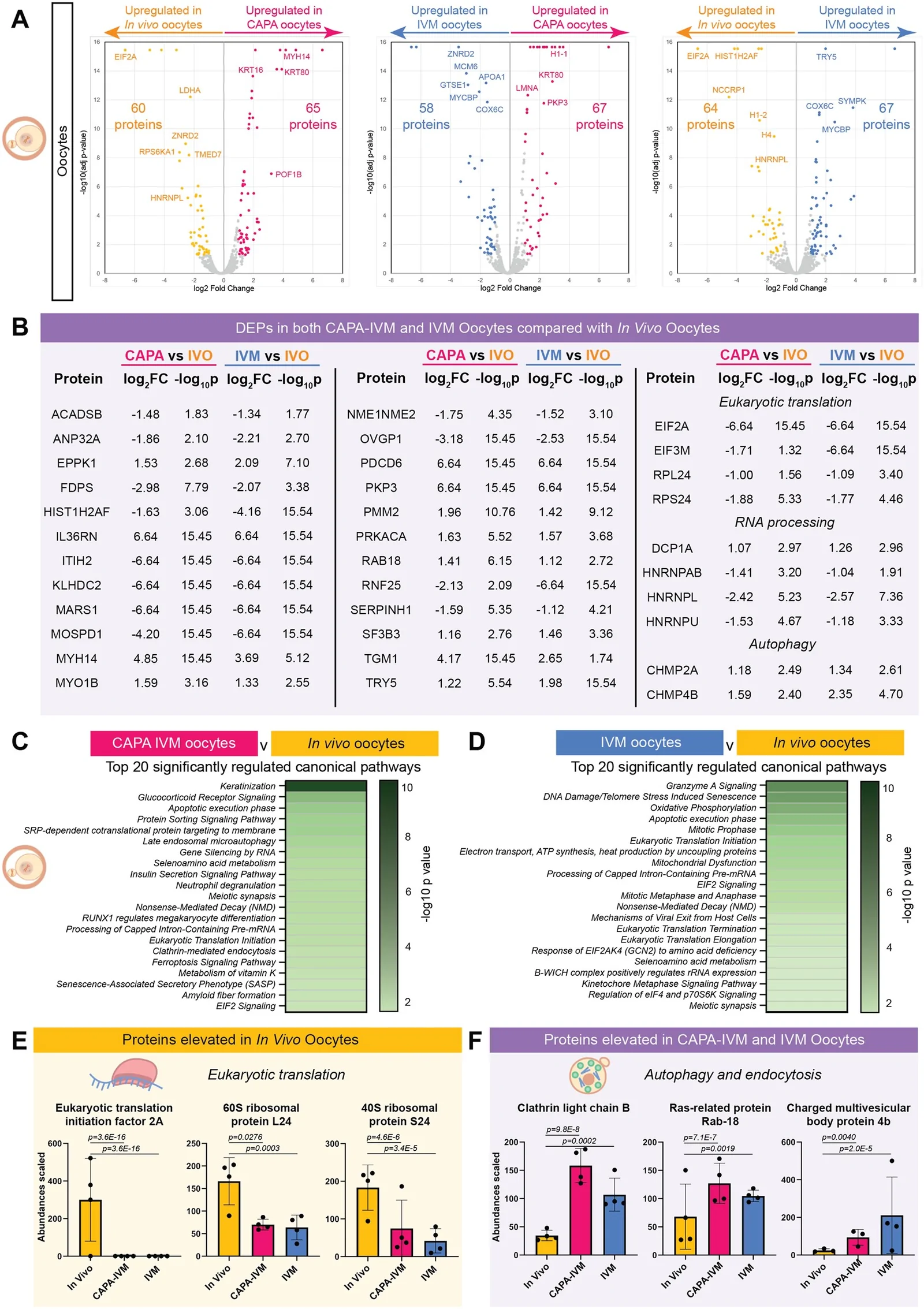
**Proteomic analysis reveals disruptions in oocyte translation machinery and endocytosis proteins.** (**A)** Volcano plots showing pairwise comparisons of differential oocyte protein expression in *in vivo* (yellow), CAPA-IVM (pink), and standard IVM (blue) treatment groups. Coloured dots indicate differentially expressed proteins in each comparison and grey dots indicate proteins below the log2FC ± 1, *P*-value ≤ 0.05 cutoff. (**B**) All proteins found to be consistently differentially expressed with log2 fold-change and − log10 *P*-values of CAPA-IVM matured MII oocytes and standard IVM matured MII oocytes compared with *in vivo* matured MII oocytes (IVO). Bar charts showing the top 20 enriched canonical pathways based on oocyte differential protein expression between (**C**) CAPA-IVM matured MII oocytes and *in vivo* matured MII oocytes, and (**D**) standard IVM matured MII oocytes and *in vivo* matured MII oocytes. Shades of green indicate − log10 *P*-value generated from Ingenuity Pathway Analysis software. Scaled proteomic abundances for individual proteins related to biological pathway elevated in (**E**) *in vivo* oocytes and (**F**) *in vitro* matured oocytes (n = 4, abundance ratio-adjusted *P*-values and mean ± SD). Icons used in the figure were generated in Biorender. Gilchrist, R. (2026) https://BioRender.com/884hwez. CAPA-IVM, capacitation-IVM; IVM, in vitro maturation; IVO, *in vivo* matured oocytes; MII, metaphase II.

Focusing on the *in vivo* and *in vitro* differential expression analysis, within the proteins associated with eukaryotic translation, eukaryotic initiation factor 2A (fold-change [FC] CAPA vs *in vivo* = −6.64, [FC] IVM vs *in vivo* = −6.64), 60S ribosomal protein L24 ([FC] CAPA vs *in vivo* = −1.00, [FC] IVM vs *in vivo* = −1.09), and 40S ribosomal protein S24 ([FC] CAPA vs *in vivo* = −1.88, [FC] IVM vs *in vivo* = −1.77) were all elevated in *in vivo* oocytes, with significantly lower levels in CAPA-IVM and IVM oocytes (Fig. 4E). In contrast, vesicular proteins including clathrin light chain B ([FC] CAPA vs *in vivo* = 1.38, [FC] IVM vs *in vivo* = 0.94), Ras-related protein ([FC] CAPA vs *in vivo* = 1.41, [FC] IVM vs *in vivo* = 1.12), and charged multivesicular body protein 4B ([FC] CAPA vs *in vivo* = 1.59, [FC] IVM vs *in vivo* = 2.35) were highly expressed in CAPA-IVM and IVM oocytes, with lower abundances found in *in vivo* oocytes (Fig. 4F). To confirm the differential expression of these target proteins across *in vivo*, CAPA-IVM and IVM oocytes, we assessed localization of a selection of these proteins within the oocyte using immunocytochemistry.

Eukaryotic initiation factor 2A (EIF2A) showed punctate expression throughout the cytoplasm of mouse oocytes, irrespective of maturation method (Fig. 5A). Expression was also observed within the polar body of all oocytes. The ribosomal subunit protein RPL24 was expressed in a diffuse pattern across the cytoplasm and polar body, with increased co-localization with the oocyte spindle (Fig. 5B). Showing similar spindle co-localization, the endocytic protein clathrin B (CLTB) was distributed primarily to the oocyte membrane, with strong localization to the oocyte spindle in all three treatment groups (Fig. 5C). Quantification of integrated density fluorescence intensity revealed a 27% decrease in expression of EIF2A in standard IVM oocytes (but not in CAPA-IVM oocytes), when compared to *in vivo* oocytes (*P* = 0.0025, Fig. 5D). This trend was mirrored in the quantification of RPL24 intensity, where IVM oocytes, but not CAPA-IVM oocytes, demonstrated a 23% decrease in protein expression against *in vivo* oocytes (*P* = 0.0003, Fig. 5E). In contrast, CLTB was elevated by 43% in CAPA-IVM oocytes when compared to *in vivo* oocytes (*P* = 0.0042, Fig. 5F), with no significant differences observed between other treatments.

**Figure 5.**
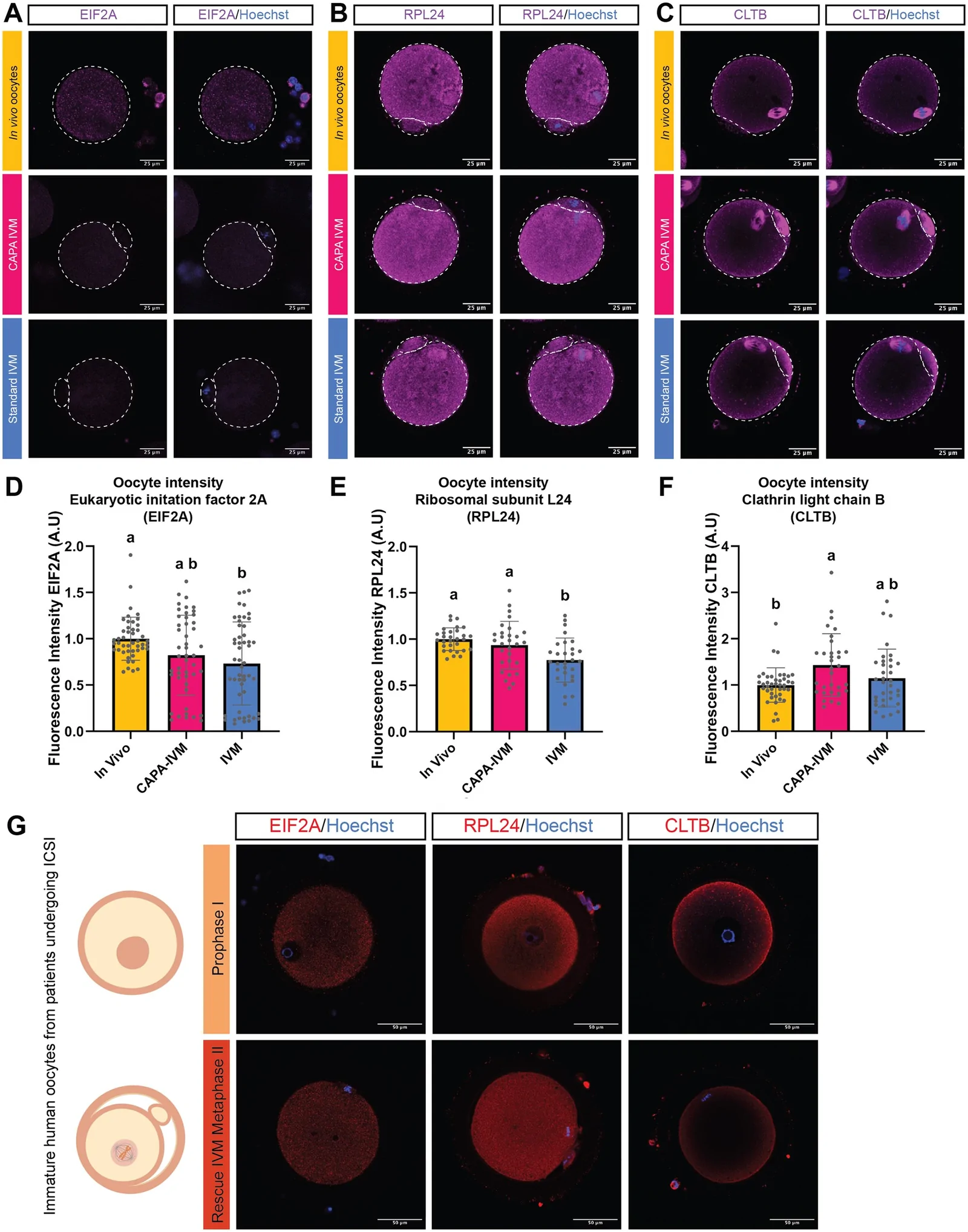
**Translation machinery and endocytosis proteins are dysregulated in *in vitro* matured oocytes.** (**A**) Eukaryotic initiation factor 2A (EIF2A, magenta), (**B**) ribosomal protein L24 (RPL24, magenta), and (**C**) clathrin light chain B (CLTB, magenta) immunocytochemistry in mouse MII oocytes following *in vivo* maturation (yellow), CAPA IVM (pink), and standard IVM (IVM; blue). Nuclei were counterstained with Hoechst (blue). Images taken at 40× magnification and scale bar denotes 25 µm. Fluorescence intensity of (**D**) EIF2A, (**E**) RPL24, and (**F**) CLTB was quantified in mouse oocytes from MII COCs following *in vivo* maturation (yellow), CAPA IVM (pink), and standard IVM (IVM; blue). Integrated density values were taken from individual COCs and normalized to the *in vivo* control mean intensity of each biological replicate (dots). Data are presented as the mean of three replicates ± SEM. Statistical analyses were performed using one-way ANOVA followed by Tukey’s *post hoc* tests. Bars with no common superscripts are significantly different (*P* < 0.05). (**G**) Immunofluorescent staining for translation machinery (RPL24 and EIF2A) and endocytosis (CLTB) in human prophase I (GV) and metaphase II oocytes. Nuclei were counterstained with Hoechst (blue). Images taken at 40× magnification and scale bar denotes 50 µm. A.U., arbitrary units; CAPA-IVM, capacitation-IVM; CLTB, clathrin light chain B; COCs, cumulus–oocyte complexes; EIF2A, eukaryotic initiation factor 2A; GV, germinal vesicle; IVM, in vitro maturation; MII, metaphase II; RPL24, ribosomal protein L24.

### Proteins altered in low competence mouse oocytes are conserved in discarded human oocytes

With translation and vesicle pathway changes observed in mouse CAPA-IVM and standard IVM, we next assessed whether these differentially expressed proteins are conserved in poor-quality human GV and MII oocytes using immunocytochemistry (Fig. 5G). Of the human GV oocytes, 51.3% matured using rescue-IVM to obtain MII oocytes for fluorescent analysis (data not shown). Staining for EIF2A and RPL24 revealed conservation of these proteins in human oocytes, with EIF2A showing punctate staining across the cytoplasm and RPL24 showing diffuse staining throughout the oocyte cytoplasm (Fig. 5G). No expression of either EIF2A or RPL24 was observed within the nucleolus of GV oocytes (Fig. 5G). Following rescue-IVM, RPL24 and EIF2A were both expressed in MII oocytes, with staining across the cytoplasm and around the spindle, with some expression also observed in the polar body (Fig. 5G). The endocytosis protein CLTB was localized to the oocyte periphery in GV oocytes, with some punctate staining within the cytoplasm (Fig. 5G). This expression pattern was consistent in MII oocytes (Fig. 5G). Combined, these data illustrate that key molecular pathway members EIF2A, RPL24, and CLTB identified in our mouse proteomic analysis are also present in human oocytes, highlighting the potential relevance of our developmental competence models to the human oocyte and clinical maturation strategies.

### Proteomic changes in *in vitro* cumulus cells reflects dysregulation in steroidogenic and metabolic pathways

In addition to assessing the oocyte proteome, we also profiled proteomic changes in cumulus cell proteins arising as a consequence of oocyte maturation mode. As with oocytes, pairwise differential expression comparisons revealed that a small proportion of the total identified proteins were differentially expressed across the three comparisons (9.0% CAPA vs *in vivo*, 6.0% CAPA vs IVM, and 7.4% IVM vs *in vivo*, log2FC of ± 1 and *P*-value ≤ 0.05, Fig. 6A, Supplementary Data File S5). We utilized the pattern-based approach to investigate differentially expressed proteins that were dysregulated in the same direction in CAPA and *in vivo* cumulus cells compared to the IVM group (log2FC of ± 1, *P*-value ≤ 0.05, Supplementary Fig. S3 and Supplementary Data File S6). This revealed 29 proteins associated with cell cycle progression and cytoskeletal remodelling (Supplementary Fig. S3). In contrast, we identified a higher number of differentially expressed proteins that were altered in the same direction in CAPA-IVM and standard IVM cumulus cells (log2FC of ± 1, *P*-value ≤ 0.05, Supplementary Data File S6). Across the three maturation systems, 125 proteins were differentially expressed in both CAPA-IVM and IVM groups relative to the *in vivo* group (Fig. 6B). This list included proteins associated with keratinization (11 keratin proteins), which may be associated with sample contamination. The list also included a number of solute carrier proteins (SLC22A6, SLC25A30, SLC6A6, SLC7A5, and SLC7A8) (Fig. 6B). To further refine our list of proteins altered *in vitro*, pathway analysis of the pairwise comparisons using IPA was performed. This revealed that the most significantly regulated canonical pathway was the response to cytosolic calcium (Fig. 7A and B). Given that calcium signalling is downstream of FSH, LH, and EGF signalling in cumulus cells during oocyte maturation and fertilization (O’Donnell *et al.*, 2004; Chen *et al.*, 2013, 2024), we looked for other novel pathways consistently altered in both comparisons. Significant canonical pathways across both comparisons included steroid biosynthesis, detoxification of reactive oxygen species, serine biosynthesis, glucocorticoid receptor signalling, and cholesterol homeostasis (Fig. 7A and B).

**Figure 6.**
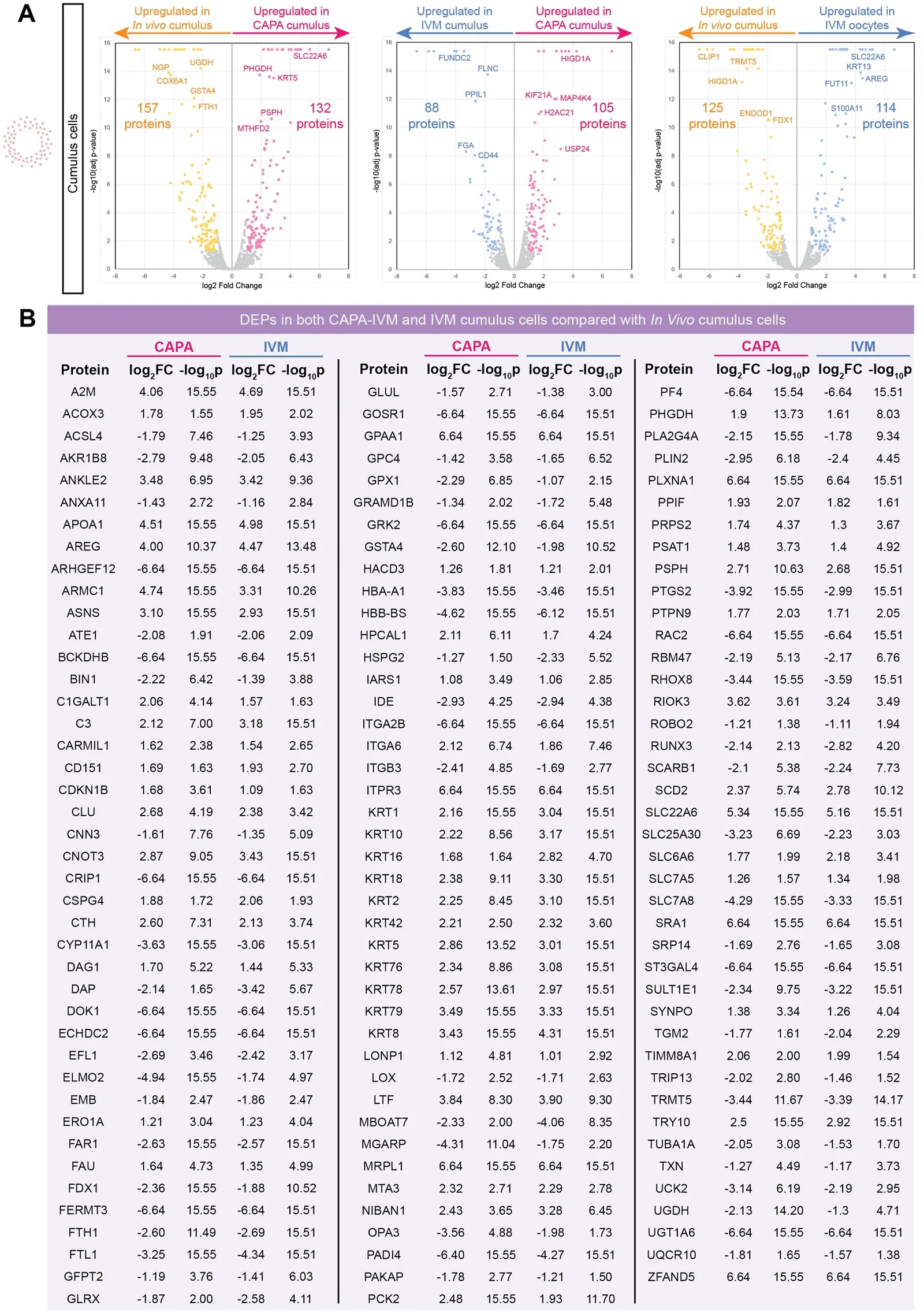
**Global proteomic alterations in cumulus cells across oocyte maturation systems.** (**A**) Volcano plots showing pairwise comparisons of differential cumulus cell protein expression in *in vivo* (yellow), CAPA-IVM (pink), and IVM (blue) treatment groups. Coloured dots indicate differentially expressed proteins in each comparison and grey dots indicate proteins below the log2FC ± 1, *P*-value ≤ 0.05 cutoff. (**B**) All proteins found to be consistently differentially expressed with log2 fold-change and − log10 *P*-values of cumulus cells from CAPA-IVM and IVM maturation groups, compared with *in vivo* matured cumulus cells (IVO). Icons used in the figure were generated in Biorender. Gilchrist, R. (2026) https://BioRender.com/884hwez. CAPA-IVM, capacitation-IVM; IVM, in vitro maturation; IVO, *in vivo* matured oocytes.

**Figure 7.**
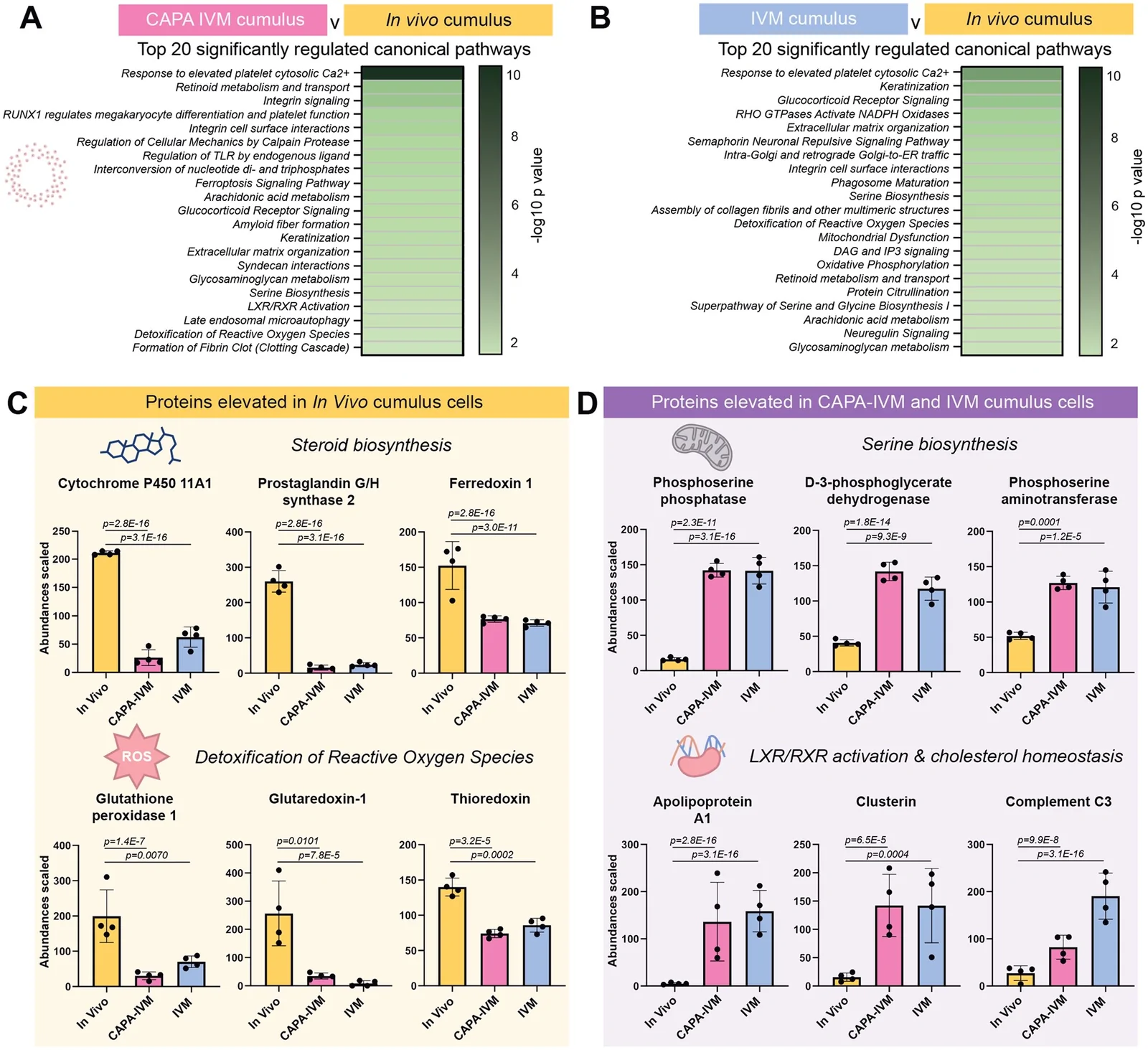
**Alterations in key biological processes underpin proteomic differences in *in vitro* matured cumulus cells.** Bar charts showing the top 20 enriched canonical pathways based on cumulus cell differential protein expression between (**A**) CAPA-IVM matured MII COCs and *in vivo* matured MII COCs, and (**B**) IVM matured MII COCs and *in vivo* matured MII COCs (IVO). Shades of green indicate − log10 *P*-value generated from Ingenuity Pathway Analysis software. Scaled proteomic abundances for individual proteins related to biological pathway elevated in (**C**) *in vivo* cumulus cells and (**D**) *in vitro* matured cumulus cells (n = 4, abundance ratio-adjusted *P*-values and mean ± SD). Icons used in the figure were generated in Biorender. Gilchrist, R. (2026) https://BioRender.com/884hwez. CAPA-IVM, capacitation-IVM; COCs, cumulus–oocyte complexes; IVM, in vitro maturation; IVO, *in vivo* matured oocytes; MII, metaphase II.

Proteins associated with these pathways showing increased expression in *in vivo* cumulus cells included cytochrome P450 11A1 (fold-change [FC] CAPA vs *in vivo* = −3.63, [FC] IVM vs *in vivo* = −3.06), prostaglandin G/H synthase 2 ([FC] CAPA vs *in vivo* = −3.92, [FC] IVM vs *in vivo* = −2.99), and ferredoxin 1 ([FC] CAPA vs *in vivo* = −2.36, [FC] IVM vs *in vivo* = −1.88): all key proteins involved in the critical function of steroid biosynthesis in cumulus cells (Fig. 7C). Additionally, proteins associated with cellular anti-oxidant response, glutathione peroxidase 1 ([FC] CAPA vs *in vivo* = −2.29, [FC] IVM vs *in vivo* = −1.07), glutaredoxin 1 ([FC] CAPA vs *in vivo* = −1.87, [FC] IVM vs *in vivo* = −2.58), and thioredoxin ([FC] CAPA vs *in vivo* = −1.87, [FC] IVM vs *in vivo* = −2.58), showed significantly higher expression in *in vivo* cumulus cells compared to CAPA-IVM and IVM cumulus cells (Fig. 7C). Conversely, proteins associated with serine and cholesterol biosynthesis were aberrantly elevated in CAPA-IVM and IVM cumulus cells when compared to *in vivo* cumulus cells (Fig. 7D). These proteins included phosphoserine phosphatase ([FC] CAPA vs *in vivo* = 2.71, [FC] IVM vs *in vivo* = 2.68), D-3 phosphoglycerate dehydrogenase ([FC] CAPA vs *in vivo* = 1.90, [FC] IVM vs *in vivo* = 1.61), phosphoserine aminotransferase ([FC] CAPA vs *in vivo* = 1.48, [FC] IVM vs *in vivo* = 1.40), apolipoprotein A1 ([FC] CAPA vs *in vivo* = 4.51, [FC] IVM vs *in vivo* = 4.98), clusterin ([FC] CAPA vs *in vivo* = 2.68, [FC] IVM vs *in vivo* = 2.38), and complement C3 ([FC] CAPA vs *in vivo* = 2.12, [FC] IVM vs *in vivo* = 3.18).

Given that both steroid production and metabolic homeostasis are essential components of cumulus cell function, particularly during oocyte maturation (Richani *et al.*, 2021; Chen *et al.*, 2023), we then focused on characterizing target proteins within the cumulus cells in each maturation strategy using immunocytochemistry (Fig. 8A and C). Ferredoxin 1 (FDX1) was intensely expressed in the cytoplasm of expanded *in vivo* cumulus cells, co-localizing with the actin cytoskeleton stain phalloidin (Fig. 8A). In CAPA and IVM cumulus cells, which demonstrate a decreased ability to expand in culture, FDX1 expression was variable, where some but not all sections of the cumulus cell clusters showed high expression (Fig. 8A). Quantification of all three groups revealed a significant decline in fluorescence intensity *in vitro*, with an 81% decrease in CAPA-IVM cumulus cells and an 82% decrease in standard IVM cumulus cells, when compared to *in vivo* cumulus cells (Fig. 8B). This analysis confirmed the decline in FDX1 protein levels observed by mass spectrometry, validating altered steroid biosynthesis in cumulus cells matured *in vitro* (Fig. 7C). We then examined D-3 phosphoglycerate dehydrogenase (PHGDH) using the same approach and found low expression of PHGDH in *in vivo* cumulus cells (Fig. 8C). Levels of PHGDH were elevated in CAPA-IVM cumulus cells throughout the nucleus and cytoplasm, with variable expression observed in standard IVM cumulus cells (Fig. 8D). CAPA-IVM cumulus cell intensity levels of PHGDH were increased by 23% and 54% compared to *in vivo* and standard IVM cumulus cells, respectively, which may indicate that CAPA-IVM cumulus cells have a higher requirement for components of the serine biosynthesis pathway, while expression of PHGDH decreased by 32% in IVM cumulus cells when compared to *in vivo* cumulus cells (Fig. 8D). Collectively, our results provide links that proteomic alterations are a critical component of oocyte quality through mechanisms involving both the oocyte and the accompanying cumulus cells.

**Figure 8.**
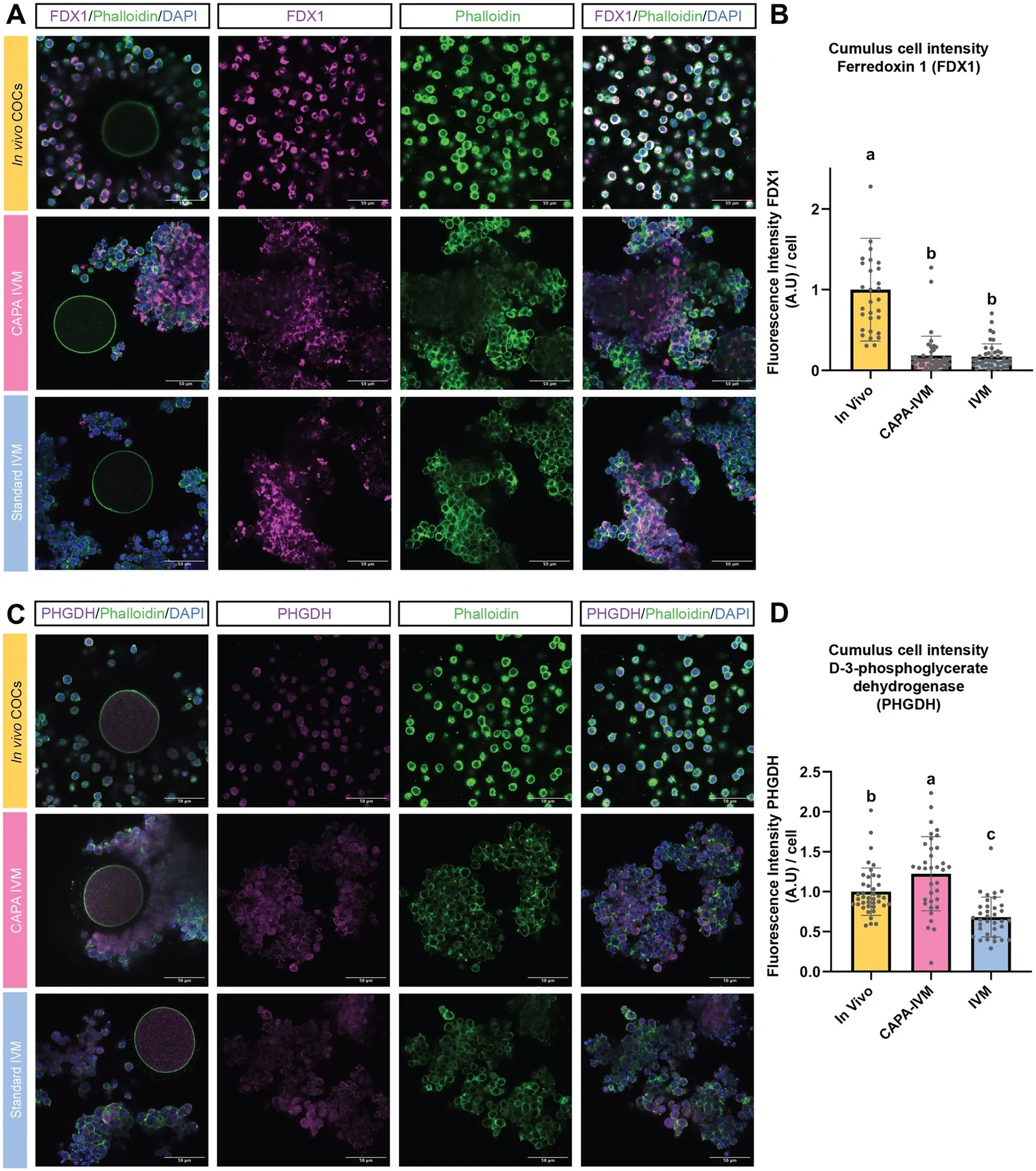
**Mitochondrial protein FDX1 and serine biosynthesis enzyme PHGDH are dysregulated in *in vitro* matured cumulus cells.** (**A**) Ferredoxin 1 (FDX1, magenta) and (**C**) D-3-phosphoglycerate dehydrogenase (PHGDH, magenta) immunocytochemistry colocalized with phalloidin (green) in MII COCs following *in vivo* maturation (yellow), CAPA-IVM (pink), and standard IVM (IVM; blue). Nuclei were counterstained with Hoechst (blue). The first column shows intact cumulus–oocyte complexes, with the subsequent columns showing representative images of cumulus cells. Images taken at 40× magnification and scale bar denotes 50 µm. Fluorescence intensity of (**B**) FDX1 and (**D**) PHGDH was quantified in cumulus cells from MII COCs following *in vivo* maturation (yellow), CAPA-IVM (pink), and standard IVM (IVM; blue). Integrated density values were taken from individual COCs, normalized by cell count and normalized to the *in vivo* control mean intensity of each biological replicate (dots). Data are presented as the mean of three replicates ± SEM. Statistical analyses were performed using one-way ANOVA followed by Tukey’s *post hoc* tests. Bars with no common superscripts are significantly different (*P* < 0.05). A.U., arbitrary units; CAPA-IVM, capacitation-IVM; COCs, cumulus–oocyte complexes; FDX1, ferredoxin 1; GV, germinal vesicle; IVM, in vitro maturation; MII, metaphase II; PHGDH, d-3-phosphoglycerate dehydrogenase.

## Discussion

Oocyte quality is a critical foundation for reproductive success. Despite information from ART and decades of research in multiple species, we still lack detailed knowledge of the complex biological processes that underpin oocyte quality, and thus future potential to support healthy embryo development. In this study, we assessed the molecular pathways underpinning developmental competence across mouse MII cumulus–oocyte complexes, that had undergone clinically relevant maturation protocols, and checked conservation of target proteins in human oocytes. Proteomic profiling revealed global alterations in both oocytes and cumulus cells between high- and low-competence mouse COCs, revealing a plethora of novel biological processes that may contribute to oocyte developmental competence. Orthogonal approaches confirmed aberrant changes to translation machinery and endocytosis proteins in oocytes following CAPA-IVM and IVM, with more profound changes in cumulus cell pathways including steroid, serine, and cholesterol biosynthesis proteins. Combined, these data shed new light on the mechanisms contributing to the decreased developmental competence seen *in vitro*, which has important clinical and biological consequences for our understanding and assessment of oocyte quality.

The focus of clinical ART improvement has predominately centred on optimizing ovarian stimulation using gonadotrophins (Schouten *et al.*, 2025). For the subset of ART patients who are particularly sensitive to gonadotrophins, for example women with polycystic ovarian syndrome (PCOS), IVM represents an alternative, safe, and low-intervention treatment option (Gilchrist and Smitz, 2023). Recently, the shift towards revisiting IVM protocols and creating new reproductive technologies has been aided by the surge of basic knowledge in oocyte and ovarian biology. CAPA-IVM, a biphasic form of IVM, relies on CNP regulating meiotic arrest through signalling from the cumulus cells (Sanchez *et al.*, 2019). CAPA-IVM emerged from a number of differing pre-IVM systems, designed to improve oocyte quality through maintained oocyte–cumulus cell communication and extended time for the accumulation of important molecular machinery (Gilchrist *et al.*, 2024). In this study, we modelled our CAPA-IVM and standard IVM treatment timings on established clinical IVM protocols (Vuong *et al.*, 2020a; De Vos *et al.*, 2025), where mice received minimal stimulation in the form of a short 23 h PMSG priming, generating intermediate and moderate-competence oocytes, respectively (Nogueira *et al.*, 2003; Zhao *et al.*, 2020). While we did observe the expected developmental competence gap between our *in vivo* matured oocytes and our *in vitro* models (∼40% change in blastocyst rate), surprisingly, we did not observe a statistical difference in embryo outcomes between CAPA-IVM and standard IVM, as we and others have previously shown in mice and humans (Sánchez *et al.*, 2017; Vuong *et al.*, 2020b; Zhao *et al.*, 2020). This guided our proteomics analysis to focus on the differentially expressed proteins between *in vivo*- and *in vitro*-matured COCs, to identify potential pathways that could be targeted to improve oocyte developmental competence *in vitro* within these clinically relevant pre-IVM and IVM systems. Of note, our proteomics data did reveal changes in identified proteins and differences in expression levels between CAPA-IVM and standard IVM cumulus–oocyte complexes that may not be reflected in the blastocyst rate. This proteomics finding is in line with previous studies revealing molecular alterations in CAPA-IVM mouse oocytes (Zhao *et al.*, 2020; Akin *et al.*, 2021). Of greater clinical interest are the substantial proteomic differences observed between high competence oocytes matured *in vivo*, compared to lower competence oocytes matured *in vitro* by either CAPA-IVM or standard IVM. While CAPA-IVM and standard IVM are well-suited to certain patient populations, such as those with PMOS, the cumulative live birth rate following CAPA-IVM remains lower than conventional IVF (Nguyen *et al.*, 2022). With the increased uptake and emerging interest in pre-IVM systems, particularly CAPA-IVM, worldwide, there exists an opportunity to further capitalize on reproductive biology research to precisely enhance *in vitro* protocols, in addition to targeting meiotic arrest.

The proteomic dataset generated in this manuscript provides an unbiased approach to assessing pathways that might dictate oocyte developmental competence. Our dataset supplements other mouse studies analysing the metabolome (Chen *et al.*, 2023), transcriptome (Ma *et al.*, 2013; Vigone *et al.*, 2013; Gao *et al.*, 2017), epigenome (Wasserzug Pash *et al.*, 2022), proteome (Richani *et al.*, 2024), secretome (Fiorentino *et al.*, 2024), active translation (Dvoran *et al.*, 2024), and the chromatin landscape (Zhu *et al.*, 2025) of mouse oocytes and/or cumulus cells. Given that the mature oocyte is largely considered transcriptionally quiescent, proteomic analysis provides the link between the stored mRNA of the oocyte and the dynamic actions of molecular machinery in actioning necessary cellular processes. Nevertheless, consolidating our proteomics dataset with these previously published studies of *in vivo* and IVM mouse oocytes will create a more comprehensive network of oocyte developmental competence at the MII stage.

Paired analyses of the oocyte and its corresponding cumulus cells are also critical to understanding developmental competence. Not only do the oocytes produce and store biochemical components themselves, but cumulus cells will deplete their stores and donate metabolites, small molecules and possibly even RNA to the oocyte (Macaulay *et al.*, 2014; Richani *et al.*, 2019; Doherty *et al.*, 2025). Analysing both the oocyte and cumulus cells separately in future studies will allow for in-depth interrogation of the substrates donated to the oocyte across the course of IVM, although such research will remain difficult in human oocytes. However, given the conservation already observed across species for some other oocyte-specific molecular processes (Pek, 2025), other aspects of developmental competence are likely to be evolutionarily essential.

The field of oocyte developmental competence is rapidly expanding (for detailed reviews, see Conti and Franciosi, 2018; Richani and Gilchrist, 2022; Tan and Dunning, 2022). In addition, it is now appreciated that both nuclear and cytoplasmic maturation are inextricably linked and must be synchronized to ensure the best gamete potential (Eppig, 1996). Within the concept of developmental competence, the oocyte holds many unusual features, for example, the presence of a midbody cap (Jung *et al.*, 2023), exit from electron transport chain dormancy (Rodríguez-Nuevo *et al.*, 2022), degradative waste processes (Zaffagnini *et al.*, 2024), and cytoplasmic lattices for protein storage (Jentoft *et al.*, 2023; Williams *et al.*, 2025): all key components of a mature oocyte competent for fertilization. Within our proteomic lists, oocyte proteins involved in zona pellucida formation (ZP1–ZP3), bi-directional communication (GDF9 and BMP15), cytoplasmic scaffolding (PADI6), and vesicular encapsulation (RUFY1) are all consistently expressed across *in vivo*, CAPA-IVM, and IVM maturation strategies. This illustrates that oocytes matured by all three methods would be deemed as high-quality by all previous demonstrations of high developmental competence, thus highlighting the need for new indicators of gamete quality within a mature oocyte.

To achieve developmental competence, oocytes must remodel their proteome to not only acquire the maternal transcripts and proteins to last through the maternal-zygotic transition but must also degrade proteins that are no longer required. Specific maternal transcripts are targeted and degraded across the GV-to-MII transition, including ribosomal protein subunits (Su *et al.*, 2007). Our proteomics data showing alterations in eukaryotic translation proteins could be explained by either of two lines of reasoning: (i) that aberrant degradation of eukaryotic translation factor and ribosomal protein subunits occur in CAPA-IVM and IVM oocytes resulting in lower protein abundances, or (ii) that these proteins are already lower in abundance due to the shortened ovarian stimulation period, and thus show lower abundance following CAPA-IVM and IVM. Future interventions including manipulations of ribosomal proteins will enable us to functionally interrogate these questions to establish the involvement of these mechanisms in oocyte quality and embryo outcomes. Combined, our data suggest that promoting the appropriate removal of transcripts and machinery is a critical requirement within pre-IVM and will ensure integrity of the oocyte translational programme.

In addition to maternal transcript degradation, redundant proteins must also be tagged and processed for waste removal or amino acid recycling. Oocytes have developed unusual vesicular strategies, termed endo-lysosomal vesicular assemblies (ELVAs) to sequester protein waste within the oocyte, observed in both mice and humans (Zaffagnini *et al.*, 2024, 2025). In this study, proteins involved with autophagy and endocytosis (CLTB, RAB18, and CHMP4A) were differentially upregulated in CAPA-IVM and IVM oocytes, when compared to *in vivo* oocytes, suggesting an accumulation of vesicles and their contents *in vitro* (Fig. 4E). While the identification of ELVAs is recent, one study assessing macroautophagy found an accumulation of LAMP1-positive vesicles in the GV oocytes of reproductively aged mice, which are known to have poor oocyte quality (Peters *et al.*, 2021). Importantly, many proteins involved in vesicular processes, including clathrin and dynamin, also co-localize to the oocyte spindle (Mihalas *et al.*, 2020; Camia *et al.*, 2025). In this study, we observed no clear change in the localization of clathrin light chain B to the oocyte membrane and spindle within *in vivo*, CAPA-IVM, or IVM oocytes (Fig. 5C). However, closer analysis of clathrin localization and intensity on the oocyte spindle and across spindle assembly using live imaging may reveal changes across the GV-to-MII transition in CAPA-IVM and IVM oocytes.

Translating results from mouse models into the human clinical setting presents a challenge, given that good-quality human oocytes are exceptionally rare research material. However, assessing the conservation of molecular pathways is essential, as molecular strategies adopted by oocytes can vary between species (Pek, 2025). Recent work has highlighted proteostasis and lysosome activity are reduced across the GV-MII translation in human oocytes, in contrast to findings in mice (Zaffagnini *et al.*, 2024, 2025). Our demonstration that key eukaryotic translation and endocytosis proteins EIF2A, RPL24, and CLTB are expressed within human oocytes presents an exciting finding. While the assessment of three key proteins selected in this study is descriptive, any opportunity to compare discarded human oocytes from traditional IVF cycles with oocytes from CAPA-IVM-treated patients would confirm whether the trends observed in our mouse models hold true for human oocytes, providing new insight into the mechanisms regulating human oocyte quality.

Our proteomics data confirmed that comprehensive remodelling of the cumulus cell proteome occurs in the absence of the follicular microenvironment (Brown *et al.*, 2017). More proteins were differentially expressed in comparisons between *in vivo* cumulus cells and the *in vitro* matured cumulus cells, reflective of poor cumulus cell expansion observed *in vitro* and that the cumulus cell proteome is highly susceptible to proteomic changes once removed from the follicular microenvironment (Chow *et al.*, 2025). Regardless, established pathways including steroid production and glutathione detoxification were altered *in vitro*: an additional confirmation of their established functions in cumulus cells for producing high-quality oocytes and subsequent embryos (Maedomari *et al.*, 2007; Trapphoff *et al.*, 2016). The identification of elevated ferredoxin-1 in *in vivo* cumulus cells links changes in steroidogenic and metabolic homeostasis, as FDX1 is an iron-sulphur protein that indirectly transfer electrons from NADPH to mitochondrial cytochrome p450 enzymes (Xing *et al.*, 2023). In contrast, the increase in proteins involved in serine biosynthesis in CAPA-IVM cumulus cells suggests a possible deficiency in serine and/or glycine in the base IVM medium. Alpha-MEM contains the standard culture medium concentration of amino acids (25 mg/l serine), as do common clinical IVM media like Medicult IVM medium, which suggests serine and/or glycine deficiency may be common in clinical IVM practice: a notion which is supported by animal studies showing glycine supplementation improves IVM outcomes (Xia *et al.*, 1995; Gao *et al.*, 2023). Our data build on the expanding evidence that metabolic homeostasis and mitochondrial function in cumulus cells is essential to enhanced oocyte developmental competence (Akin *et al.*, 2021; Tan *et al.*, 2022; Chen *et al.*, 2023; Herta *et al.*, 2023; Richani *et al.*, 2024).

Investigating the molecular signature of oocyte developmental competence presents numerous biological and technical challenges. Limited access to good-quality human oocytes for research means that model species are imperative to investigate oocyte development and maturation. However, care is required when interpreting the significance of these findings in mice to mechanisms that may not be conserved in human oocytes. In addition, the recent limited uptake of CAPA-IVM and IVM into the clinic also means that access to human CAPA-IVM and IVM samples that would otherwise be discarded are sparse and difficult to obtain. In the mouse, our best indicator of developmental competence relies on generating embryos and comparing blastocyst rates, which is a blunt measure and does not reflect individual oocyte quality. In our study, our embryology results are inconsistent with the IVM literature, as we do not see a difference in developmental competence between CAPA-IVM and IVM matured oocytes (Romero *et al.*, 2016; Sanchez *et al.*, 2019; Vuong *et al.*, 2020b; Zhao *et al.*, 2020; Gong *et al.*, 2023). Increasing sample size and performing embryo transfer experiments may reveal developmental competence differences between the two systems. This study has also not addressed oocyte developmental competence in gonadotropin-free IVM oocytes, which is now a clinical reality (Vuong *et al.*, 2025). Although our IVM and CAPA-IVM mouse models closely match the human clinical systems, they require fetuin in the culture medium to overcome the mouse-specific phenomenon of zona hardening (Dietzel *et al.*, 2013). Finally, using the combination of proteomics and immunofluorescence to validate protein changes in oocytes and cumulus cells meant that not all trends were conserved, likely reflecting methodological divergence. Proteomics provides a bulk measurement of protein abundance across the entire sample, but can be limited to highly expressed proteins and may not detect lowly expressed proteins. In contrast, immunofluorescence captures spatially resolved expression within selected cells or regions. Variation between the two techniques may arise from differences in antibody specificity, signal dynamic range, sample heterogeneity, and post-translational modifications that may affect antibody recognition without altering overall protein abundance. Nonetheless, utilizing two methods for target validation provides additional confidence for the role of these proteins in promoting developmental competence. For each target assessed, at least one pairwise group comparison reflected the proteomics fold-changes observed, providing some evidence of the changes important for developmental competence. We also were unable to verify the importance of keratin proteins in our *in vitro* oocyte groups, where it is unclear whether the keratin expression is of mouse or human origin. Keratins are essential during early pre-implantation embryo development (Lim *et al.*, 2020) and may be acquired by the oocyte as a component of developmental competence, but could also be explained by the increased handling and culture periods of *in vitro* matured oocytes. Combined, these limitations provide important context for interpreting our proteomics dataset as a signature of oocyte quality.

In summary, we have compared three models of oocyte maturational competence, showing differential proteomic alterations in oocytes and cumulus cells matured *in vitro*, relative to those matured *in vivo*. Compromised translation and vesicular processes in *in vitro* oocytes, along with functional changes in metabolic and amino acid production within cumulus cells, are promising new contributors to oocyte developmental competence. As the global ART pregnancy rate plateaus (Chambers *et al.*, 2021), new insights into oocyte developmental competence hold the potential to improve clinical oocyte diagnostics, expand on current clinical maturation systems, and provide novel therapeutic options to infertile patients. With emerging pre-IVM and IVM treatment options, a strengthened understanding of oocyte developmental competence holds the potential to improve the developmental capacity of *in vitro* matured oocytes. This research builds towards the overall clinical goal of ART, to achieve high-quality oocytes and eventual offspring for patients, regardless of the mode of oocyte maturation.

## Supplementary Material

deag134_Supplementary_Data

deag134_Supplementary_Figure_S1

deag134_Supplementary_Figure_S2

deag134_Supplementary_Figure_S3

deag134_Supplementary_Table_S1

deag134_Supplementary_Table_S2

## Data Availability

The mass spectrometry proteomics data have been deposited to the ProteomeXchange Consortium via the PRIDE (Perez-Riverol *et al.*, 2025) partner repository with the dataset identifier PXD073269 https://doi.org/10.6019/PXD073269.
