# Supplementary material for: A proteomic signature of oocyte quality from models of varying oocyte developmental competence
Source: Hum Reprod. 2026 Aug 20;41(10):1741–61. doi: 10.1093/humrep/deag134 (PMC13635460; doi:10.1093/humrep/deag134)
Supplement: Supplementary file 1 [file deag134_supplementary_figure_s1.pdf]

Human Reproduction, 2026, 1–1
https://doi.org/10.1093/humrep/deag134
Supplementary Data

human reproduction

OXFORD

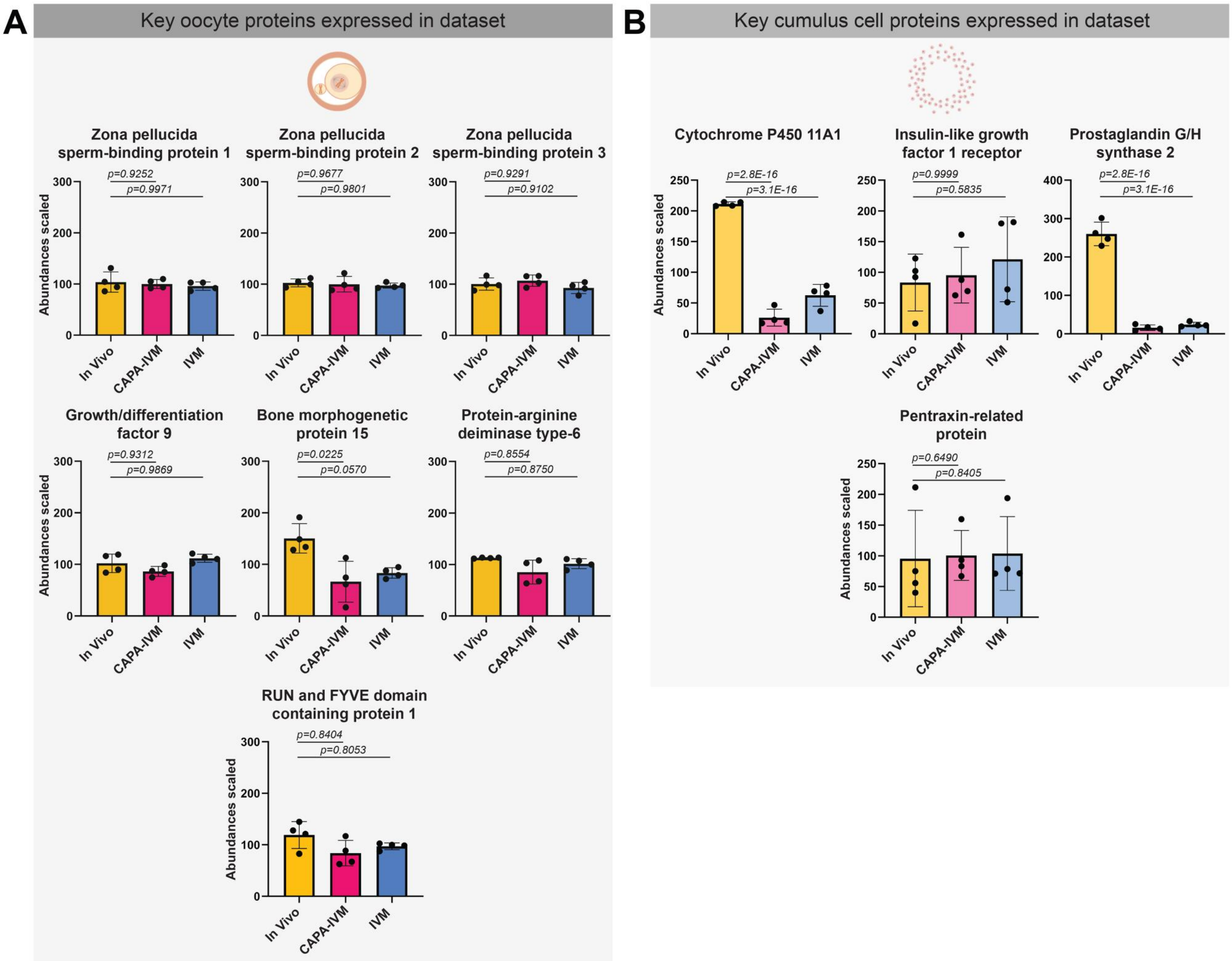

Supplementary Figure S1. Key oocyte and cumulus cell protein expression levels across models of developmental competence. Scaled proteomic abundances for individual proteins across *in vivo* (yellow), CAPA-IVM (pink), and IVM (blue) treatment groups expressed in (**A**) oocytes and (**B**) cumulus cells (n = 4, abundance ratio-adjusted *P*-values and mean ± SD). Icons used in the figure were generated in Biorender. Gilchrist, R. (2026) https://BioRender.com/884hwez. CAPA-IVM, capacitation-IVM; IVM, in vitro maturation.
