# Supplementary material for: A proteomic signature of oocyte quality from models of varying oocyte developmental competence
Source: Hum Reprod. 2026 Aug 20;41(10):1741–61. doi: 10.1093/humrep/deag134 (PMC13635460; doi:10.1093/humrep/deag134)
Supplement: Supplementary file 2 [file deag134_supplementary_figure_s2.pdf]

Human Reproduction, 2026, 1–1
https://doi.org/10.1093/humrep/deag134
Supplementary Data
human reproduction
OXFORD

A

DEPs in both CAPA-IVM and In Vivo Oocytes compared with IVM Oocytes

Protein	CAPA vs IVM log_2FC	CAPA vs IVM $-log_{10}p$	IVM vs IVO log_2FC	IVM vs IVO $-log_{10}p$
APOA1	-1.61	13.17	1.31	7.73
AURKC	6.64	15.65	-6.64	15.54
CCDC186	6.64	15.65	-6.64	15.54
CKAP2	6.64	15.65	-6.64	15.54
COX17	-2.68	8.11	1.19	2.41
COX6c	-1.53	11.85	1.56	11.13
EIF2B3	6.64	15.65	-6.64	15.54
FSCN1	6.64	15.65	-6.64	15.54
GTSE1	-2.83	13.04	2.33	6.45
GYG1	-1.25	4.54	1.06	1.85
H1-0	6.64	15.65	-6.64	15.54
H1-2	2.23	15.65	-2.47	10.58
H1-3	2.2	5.22	-2.99	7.42
H1-4	2.27	15.65	-1.82	4.37
H1-5	2.33	15.65	-2.34	15.54
H2AZ2	6.64	15.65	-6.64	15.54
H4C1, H4C2	1.15	11.35	-1.49	9.48
HID1	-6.64	15.65	6.64	15.54
HNRNPDL	1.98	15.65	-1.6	3.47
HNRNPR	6.64	15.65	-6.64	15.54
KRT77	6.64	15.65	-6.64	15.54
LBR	1.36	1.89	-1.26	1.55
LRPPRC	6.64	15.65	-6.64	15.54
MCM6	-2.94	13.84	1.68	2.60
MEA1	-1.34	3.07	1.14	1.52
MYCBP	-2.07	12.57	2.6	10.47
MYO1C	1.77	1.82	-3.05	2.58
NCCRP1	3.07	6.22	-4.53	12.20
NUP62	-1.28	1.89	2.03	2.55
OXCT1	6.64	15.65	-6.64	15.54
PALM3	6.64	15.65	-6.64	15.54
PFAS	6.64	15.65	-6.64	15.54
PFDN6	-1.31	3.95	1.63	3.89
PFKP	6.64	15.65	-6.64	15.54
PKP1	-1.27	3.88	1.42	3.18
PREP	6.64	15.65	-6.64	-6.64
RCN2	-1.71	3.94	2.02	3.36
SLC45A3	2.48	15.65	-3.94	15.54
SMC2	6.64	15.65	-6.64	15.54
SRSF7	6.64	15.65	-6.64	15.54
SWI5	-1.74	4.39	2.02	3.28
SYMPK	-2.38	7.27	3.83	11.46
TMSB10	-1.05	4.62	1.33	7.89
UQCRH	-1.24	2.05	1.67	3.47
USP30	1.51	1.74	-1.55	1.43

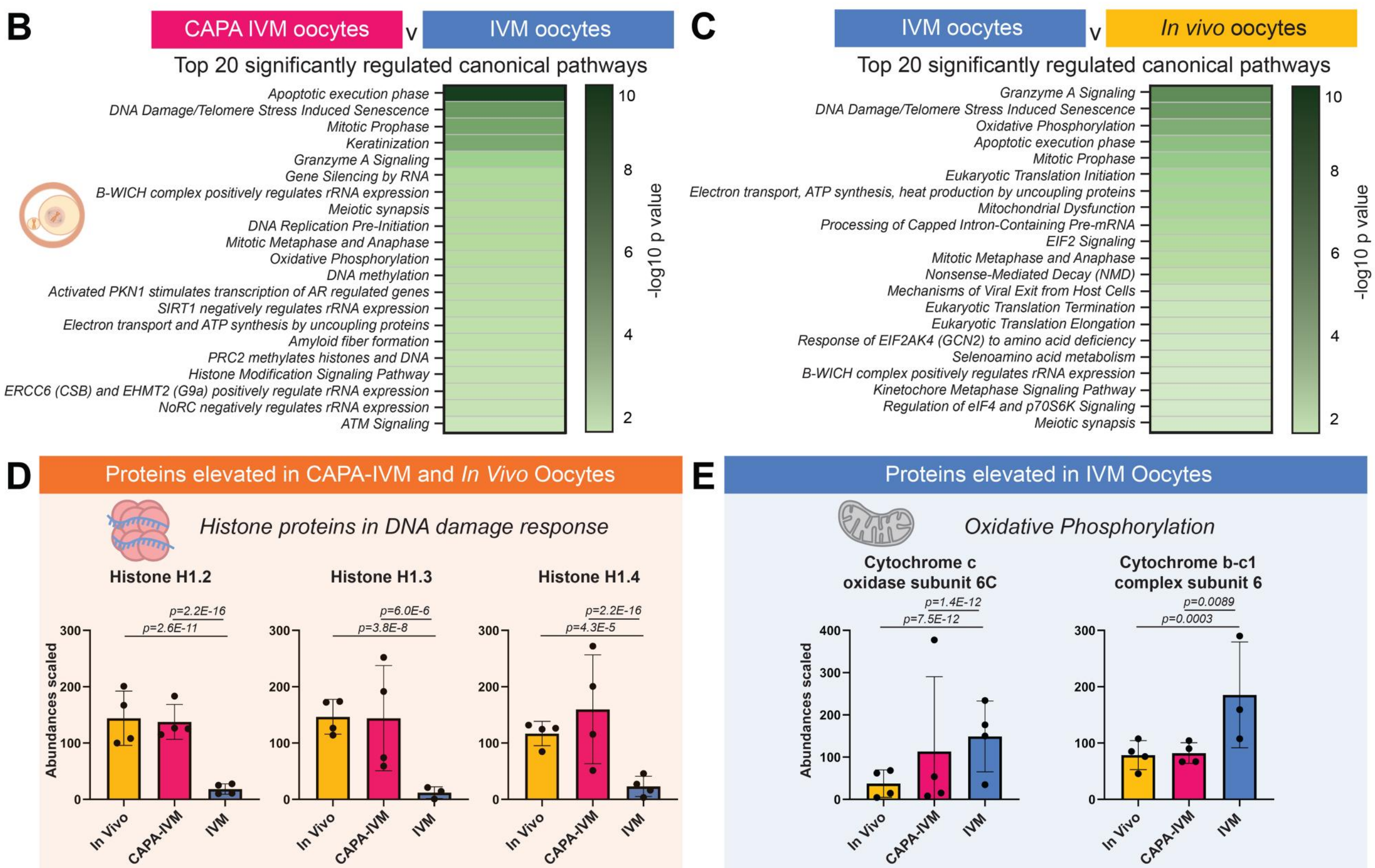

Supplementary Figure S2. Histone proteins and oxidative phosphorylation processes are changed in *in vitro* matured oocytes. (**A**) All proteins found to be consistently differentially expressed with log2 fold-change and – log10 *P*-values of CAPA-IVM matured MII oocytes and *in vivo* matured MII oocytes (IVO) compared with standard IVM matured MII oocytes (IVM). Bar charts showing the top 20 enriched canonical pathways based on oocyte differential protein expression between (**B**) CAPA-IVM matured MII oocytes and standard IVM matured MII oocytes, and (**C**) standard IVM matured MII oocytes and *in vivo* matured MII oocytes. Shades of green indicate – log10 *P*-value generated from Ingenuity Pathway Analysis software. Scaled proteomic abundances for individual proteins related to biological pathway elevated in (**D**) CAPA and *in vivo* oocytes and (**E**) IVM matured oocytes (n = 4, abundance ratio-adjusted *P*-values and mean ± SD). Icons used in the figure were generated in Biorender. Gilchrist, R. (2026) https://BioRender.com/884hwez. CAPA-IVM, capacitation-IVM; IVM, in vitro maturation; IVO, *in vivo* matured oocytes; MII, metaphase II.
