# Supplementary material for: A proteomic signature of oocyte quality from models of varying oocyte developmental competence
Source: Hum Reprod. 2026 Aug 20;41(10):1741–61. doi: 10.1093/humrep/deag134 (PMC13635460; doi:10.1093/humrep/deag134)
Supplement: Supplementary file 3 [file deag134_supplementary_figure_s3.pdf]

Human Reproduction, 2026, 1–1
https://doi.org/10.1093/humrep/deag134
Supplementary Data

human reproduction

OXFORD

A

DEPs in both CAPA-IVM and In Vivo Oocytes compared with IVM Oocytes

	CAPA vs IVM		IVM vs IVO			CAPA vs IVM		IVM vs IVO			CAPA vs IVM		IVM vs IVO	
Protein	log_2FC	$-log_{10}p$	log_2FC	$-log_{10}p$	Protein	log_2FC	$-log_{10}p$	log_2FC	$-log_{10}p$	Protein	log_2FC	$-log_{10}p$	log_2FC	$-log_{10}p$
1810009J06Rik	-1.38	5.25	2.64	10.90	FLNC	-1.81	13.74	2.45	15.51	POP1	-1.65	1.72	1.83	1.38
ADH7	-2.03	2.24	2.53	2.66	GDA	6.64	15.37	-6.64	15.51	RAB3GAP1	6.64	15.37	-6.64	15.51
AURKB	6.64	15.37	-6.64	15.51	GOLT1B	1.96	2.33	-2.63	3.56	RELCH	-1.41	1.44	1.87	1.75
AVIL	-1.70	3.70	1.56	1.60	HIGD1A	4.24	15.37	-3.75	13.18	S100A11	-1.70	4.60	3.33	11.00
BOD1L	18.2	1.35	-2.26	1.60	LCP1	6.64	15.37	-6.64	15.51	TMA7	1.98	7.69	-1.5	1.89
BTC	1.16	1.36	-1.91	2.73	MAVS	6.64	15.37	-6.64	15.51	TMEM86A	-1.08	2.00	1.64	3.97
CBR2	6.64	15.37	-6.64	15.51	NAP1L5	2.01	15.37	-2.35	15.51	TPRG1L	-1.21	1.55	2.65	4.48
CD44	-2.69	8.05	1.99	2.37	PIN1	1.79	3.57	-1.73	1.54	TRIM33	3.71	15.37	-3.49	7.65
COL6A3	-1.83	3.19	2.25	2.90	PLEC	-1.01	3.85	1.20	4.27	VAMP7	-5.84	15.37	6.64	15.51
COX4I2	2.07	9.09	-1.98	4.42	PLK1	6.64	15.37	-6.64	15.51					

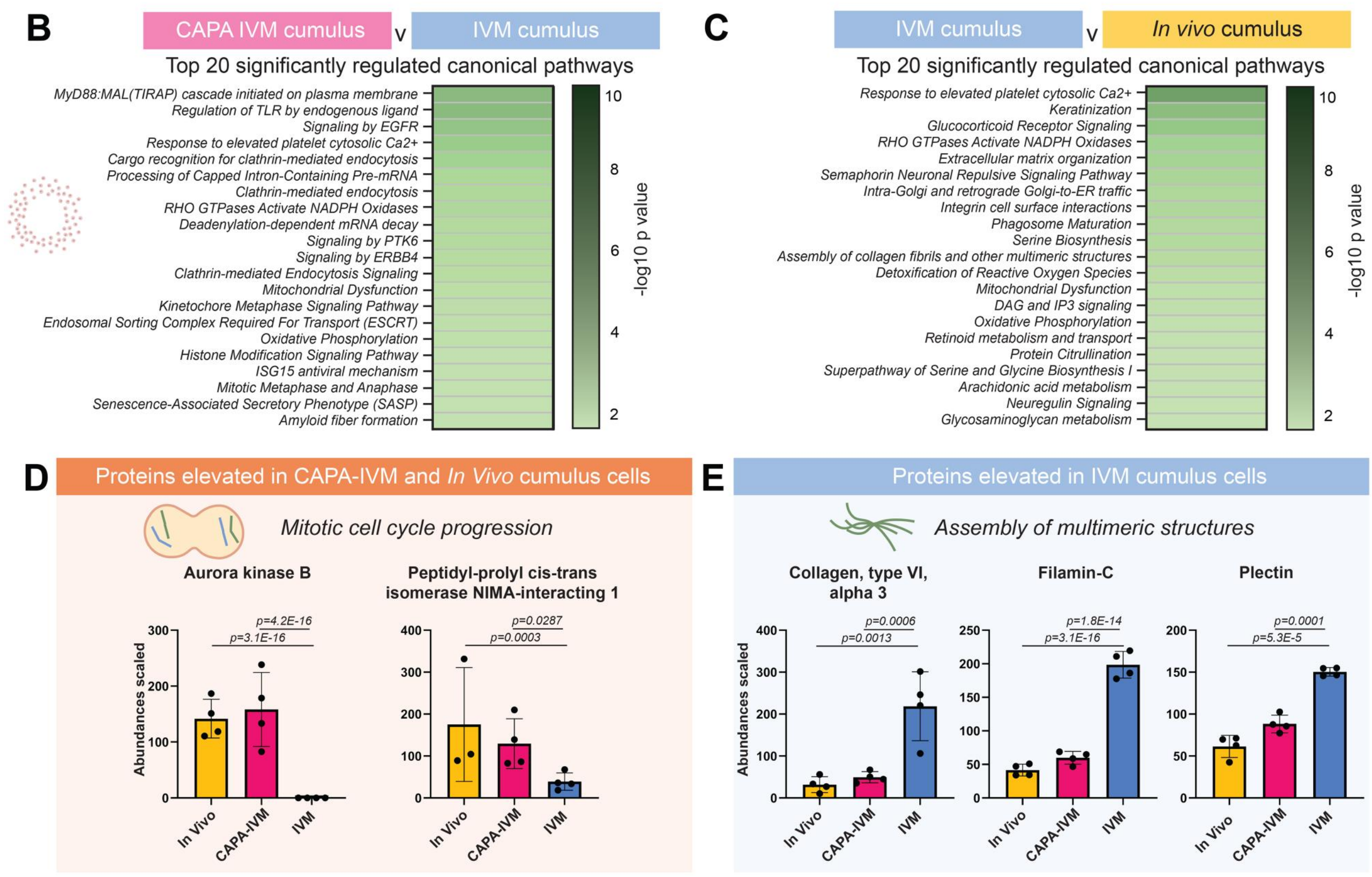

Supplementary Figure S3. Proteomic analysis reveals alterations in cell cycle and filament proteins in *in vitro* matured cumulus cells. (**A**) All proteins found to be consistently differentially expressed with log2 fold-change and – log10 *P*-values of CAPA-IVM matured MII cumulus cells and *in vivo* matured cumulus cells (IVO) compared with standard IVM matured cumulus cells (IVM). Bar charts showing the top 20 enriched canonical pathways based on cumulus cell differential protein expression between (**B**) CAPA-IVM and standard IVM cumulus cells, and (**C**) standard IVM and *in vivo* cumulus cells. Shades of green indicate – log10 *P*-value generated from Ingenuity Pathway Analysis software. Scaled proteomic abundances for individual proteins related to biological pathway elevated in (**D**) CAPA and *in vivo* cumulus cells and (**E**) IVM cumulus cells (n = 4, abundance ratio-adjusted *P*-values and mean ± SD). Icons used in the figure were generated in Biorender. Gilchrist, R. (2026) https://BioRender.com/884hwez. CAPA-IVM, capacitation-IVM; IVM, in vitro maturation; IVO, *in vivo* matured oocytes; MII, metaphase II.
