# Supplementary material for: A proteomic signature of oocyte quality from models of varying oocyte developmental competence
Source: Hum Reprod. 2026 Aug 20;41(10):1741–61. doi: 10.1093/humrep/deag134 (PMC13635460; doi:10.1093/humrep/deag134)
Supplement: Supplementary file 4 [file deag134_supplementary_table_s1.pdf]

Human Reproduction, 2026, 1–1
https://doi.org/10.1093/humrep/deag134
Supplementary Data

human reproduction
OXFORD

Supplementary Table S1. Number of cumulus–oocyte complexes (COCs) per replicate used for label-free proteomics.

Number of cumulus–oocyte complexes (COCs) per replicate			
	In vivo	CAPA-IVM	Trad-IVM
Replicate 1	277	198	198
Replicate 2	214	156	150
Replicate 3	200	106	106
Replicate 4	316	126	123
