# Supplementary material for: A proteomic signature of oocyte quality from models of varying oocyte developmental competence
Source: Hum Reprod. 2026 Aug 20;41(10):1741–61. doi: 10.1093/humrep/deag134 (PMC13635460; doi:10.1093/humrep/deag134)
Supplement: Supplementary file 5 [file deag134_supplementary_table_s2.pdf]

Human Reproduction, 2026, 1–1
https://doi.org/10.1093/humrep/deag134
Supplementary Data

human reproduction
OXFORD

Supplementary Table S2. Antibodies used in this study.

Antibody	Source/reference	Catalogue no.	RRID	Dilution (concentration)
Anti-ADX (FDX1) Rabbit Monoclonal	Abcam, Cambridge, UK	ab108257	AB_10862209	1:100 (9.28 μg/ml)
Anti-CLTB Rabbit Polyclonal	ThermoFisher Scientific, Waltham, MA, USA	PA5-109557	AB_2854968	1:200 (4.55 μg/ml)
Anti-EIF2A Rabbit Monoclonal	Abcam, Cambridge, UK	ab169528	AB_2819002	1:100 (4.16 μg/ml)
Anti-PHGDH Rabbit Polyclonal	ThermoFisher Scientific, Waltham, MA, USA	PA5-27578	AB_2545054	1:500 (1.74 μg/ml)
Anti-RPL24 Rabbit Polyclonal	ThermoFisher Scientific, Waltham, MA, USA	PA5-30157	AB_2547631	1:100 (10.20 μg/ml)

© The Author(s) 2026. Published by Oxford University Press on behalf of European Society of Human Reproduction and Embryology.
This is an Open Access article distributed under the terms of the Creative Commons Attribution License (https://creativecommons.org/licenses/by/4.0/), which permits unrestricted reuse, distribution, and reproduction in any medium, provided the original work is properly cited.
Human Reproduction, 2026, 00, 1–26
https://doi.org/10.1093/humrep/deag134
Original Article
